# HAC1 and HAF1 Histone Acetyltransferases Have Different Roles in UV-B Responses in Arabidopsis

**DOI:** 10.3389/fpls.2017.01179

**Published:** 2017-07-10

**Authors:** Julieta P. Fina, Fiorella Masotti, Sebastián P. Rius, Franco Crevacuore, Paula Casati

**Affiliations:** Centro de Estudios Fotosintéticos y Bioquímicos, Universidad Nacional de RosarioRosario, Argentina

**Keywords:** Arabidopsis, histone acetyltransferases, CBP, HAC, TAFII250, HAF, UV-B

## Abstract

Arabidopsis has 12 histone acetyltransferases grouped in four families: the GNAT/HAG, the MYST/HAM, the p300/CBP/HAC and the TAFII250/HAF families. We previously showed that *ham1* and *ham2* mutants accumulated higher damaged DNA after UV-B exposure than WT plants. In contrast, *hag3* RNA interference transgenic plants showed less DNA damage and lower inhibition of plant growth by UV-B, and increased levels of UV-B-absorbing compounds. These results demonstrated that HAM1, HAM2, and HAG3 participate in UV-B-induced DNA damage repair and signaling. In this work, to further explore the role of histone acetylation in UV-B responses, a putative function of other acetyltransferases of the HAC and the HAF families was analyzed. Neither HAC nor HAF acetyltrasferases participate in DNA damage and repair after UV-B radiation in Arabidopsis. Despite this, *haf1* mutants presented lower inhibition of leaf and root growth by UV-B, with altered expression of *E2F* transcription factors. On the other hand, *hac1* plants showed a delay in flowering time after UV-B exposure and changes in *FLC* and *SOC1* expression patterns. Our data indicate that HAC1 and HAF1 have crucial roles for in UV-B signaling, confirming that, directly or indirectly, both enzymes also have a role in UV-B responses.

## Introduction

Posttranslational modifications of histones, including acetylation, are critical mechanisms that affect different aspects of plant growth and development (Kouzarides, 2007). These modifications alter the chromatin structure, and thus have an essential role in DNA metabolism. Posttranslational modifications act consecutively or in combination in a pattern known as the “histone code”, which induces conformational changes in chromatin, therefore affecting for example the accessibility of genes to the transcriptional machinery. In particular, histone acetyltransferases regulate the acetylation of histones and other proteins such as transcription factors, affecting chromatin organization, transcriptional regulation, and DNA metabolism in general. Hyperacetylated histone lysines are associated to actively transcribed genes, while hypoacetylated histones are typically linked with transcriptionally inactive DNA (Hebbes et al., 1988; Sterner and Berger, 2000; Richards and Elgin, 2002). Acetylated lysines at the amino-terminal histone tails change histone–DNA interactions and make the DNA more accessible (Kouzarides, 2007). Thus, these changes in histone acetylation/deacetylation are essential for the regulation of gene expression (Lusser et al., 2001; Richards and Elgin, 2002; Loidl, 2004; Fuchs et al., 2006). On the other hand, the modification of the histones–DNA interactions by changes in histone acetylation can also alter DNA repair rates. For example, maize and Arabidopsis plants treated with an inhibitor of histone acetyltransferases, curcumin, previous to a UV-B treatment show increased DNA damage (Campi et al., 2012).

Key participants in histone acetylation are histone acetyltransferases (HATs), which transfer acetyl groups to the NH_3_^+^ groups of lysine residues (Roth et al., 2001). In Arabidopsis, there are 12 HATs; which are organized in four families based on sequence homology and/or its mode of action: the p300/CBP or HAC family (HAC1, HAC2, HAC4, HAC5, and HAC12); the TAFII250 or HAF family (HAF1 and HAF2); the GNAT or HAG family (HAG1, HAG2, and HAG3), and the MYST or HAM family (HAM1 and HAM2) (Pandey et al., 2002).

Regarding HATs from the p300/CBP family, Arabidopsis has 5 HACs proteins, HAC1 (At1g79000); HAC2 (At1g67220); HAC4 (At1g55970); HAC5 (At3g12980), and HAC12 (At1g16710); HAC2 has diverged earlier during evolution than the other four Arabidopsis HAC proteins (Pandey et al., 2002). Accordingly, HAC2 did not show any HAT activity in *in vitro* assays, whereas all the other 4 HACs showed HAT activity (Bordoli et al., 2001). While HAC1 specifically acetylates histone H4K14; HAC1, HAC5, and HAC12 can acetylate H3K9 amongst other Lys residues, showing broad-specificity in their activities (Earley et al., 2007). On the other hand, HAC1, HAC5, and HAC12 promote flowering regulating transcription of *FLOWERING LOCUS C* (*FLC*), a major floral repressor (Deng et al., 2007; Han et al., 2006).

Proteins of the HAF family or TAF1 proteins are part of the pre-initiation complex during transcription initiation, together with RNA polymerase II and a subset of other core transcription factors. The C-terminal bromodomain of TAF1 interacts with acetylated histones H4, H3 and H2A *in vivo;* this interaction increases TAF1 acetyltransferase activity resulting in further histone acetylation and transcription activation (Martinez, 2002; Kanno et al., 2004). Arabidopsis has two HAF genes, HAF1 or TAF1 (At1g32750) and HAF2 or TAF1b (At3g19040; Pandey et al., 2002; Lago et al., 2004). HAF2 has certain roles during plant development and mediates light responses (Bertrand et al., 2005; Benhamed et al., 2006). *haf2* mutant plants are viable but are chlorotic; while no major growth defects were observed in the *haf1* mutant line used in that same study (Bertrand et al., 2005). Interestingly, HAF1 was suggested to participate in DNA damage repair by X-rays and mitomycin C, which produce double strand breaks in DNA (Waterworth et al., 2015). However, the same *haf1* mutants were not sensitive to methyl methanesulfonate, which causes alkylation to DNA bases.

UV-B induces the formation of covalent bonds between adjacent pyrimidines in DNA, generating cyclobutane pyrimidine dimers (CPD) and pyrimidine (6–4) pyrimidone photoproducts (6-4PPs) (Friedberg et al., 1995). The formation of these dimers disturbs base pairing and blocks DNA metabolism, and results in mutations if photoproducts are not repaired (Britt, 1996). Interestingly, this radiation does not produce double strand breaks in DNA as other genotoxic agents as UV-C or X-rays (Britt, 1996). In maize and Arabidopsis, histone acetylation has been associated to UV-B responses and damage repair after exposure (Casati et al., 2006, 2008; Campi et al., 2012; Fina and Casati, 2015). Analysis of post-translational modifications of histones showed that UV-B exposed maize plants have higher acetylation of N-terminal H3 and H4 tails (Casati et al., 2008). Moreover, when maize and Arabidopsis plants were sprayed with curcumin, a histone acetylase inhibitor, before the treatment, DNA repair was reduced (Campi et al., 2012). Thus, we previously investigated the role of HATs of the MYST/HAF and the GNAT/HAG family in UV-B responses in Arabidopsis (Campi et al., 2012; Fina and Casati, 2015; Falcone Ferreyra et al., 2016). Interestingly, *ham1* and *ham2* mutants showed higher DNA damage after a 4h-UV-B-treatement (Campi et al., 2012). Therefore, the role of MYST family acetyltransferases in DNA damage repair seems to be conserved; as TIP60, the human homologue to *At*HAM1 and *At*HAM2, also participates in DNA repair, transactivating genes and acetylating H4 after DNA damage (Squatrito et al., 2006). On the other hand, *hag3* RNA interference (RNAi) transgenic plants presented a lower inhibition of plant growth by UV-B, with higher levels of UV-B-absorbing compounds and less DNA damage after UV-B exposure than WT plants (Fina and Casati, 2015). Expression of UV-B-regulated genes was increased in the absence of UV-B in *hag3* RNAi transgenic plants; thus, the increased UV-B tolerance in these plants could be the result of increased levels of proteins that participate in UV-B responses. Together, our previous data demonstrate that HAM1, HAM2 and HAG3 participate in UV-B-induced DNA damage repair and signaling.

In this work, to further explore the role of histone acetylation in UV-B responses, a putative function of other histone acetyltransferases of the p300/CBP and the TAFII250 families was analyzed in Arabidopsis. We here provide evidence that neither HAC nor HAF acetyltrasferases participate in DNA damage and repair after UV-B radiation in Arabidopsis plants. However, *haf1* mutants show lower inhibition of leaf and root growth by UV-B; while *hac1* plants have a delay in flowering time after UV-B exposure more significant than that of WT plants. Finally, we show here that several genes that are UV-B regulated in WT plants have altered expression levels in *haf1* and *hac1* plants, demonstrating crucial contributions of HAF1 and HAC1 in UV-B signaling. Together, our data provide evidence that HAF1 and HAC1, directly or indirectly, have a role in UV-B signaling.

## Materials and Methods

### Plant Material, Growth Conditions, and Irradiation Protocols

*Arabidopsis thaliana* ecotype Columbia (Col-0) and Wassilewskija (Ws) lines were used for the experiments (Supplementary Figure S1). RNAi lines were generated in the Plant Chromatin Consortium^1^. The RNAi transgenic lines (*haf1-4*: CS30866; *hac2-2*: CS30854, and *hac2-3*: CS30855) and the T-DNA insertion mutants (*ham1-1*: SALK_027726; *hac1-3*: SALK_080380C; *hac1-1*: SALK_082118C; *hac4-1*: SALK_051750C; *hac4-4*: SALK_045791C; *hac5-7*: SALK_152684C; *hac5-8*: SALK_122443C; *hac12-2*: SALK_012469C; *hac12-3*: SALK_071102C; *haf1-3*: SALK_110848; *haf2-3*: SALK_110029; *haf2-4*: SALK_038282C) were obtained from the Arabidopsis Biological Resource Center (ABRC, Columbus, OH, United States). *haf1-3* plants express a truncated mRNA lacking the region encoding the bromodomain (Waterworth et al., 2015); all the other T-DNA insertion mutants used showed undetectable or decreased transcript levels; while RNAi transgenic lines showed decreased mRNA levels as shown in Supplementary Figure S2.

Arabidopsis plants were sown directly on soil and placed at 4°C in the dark. After 3 days, pots were relocated to a greenhouse and plants were grown at 22°C under a 16 h/8h light/dark photoperiod. UV treatments were done in a growth chamber with supplemental visible light (100 μEm^-2^s^-1^; 16 h/8h light/dark photoperiod). For most experiments, plants were irradiated with UV-B lamps for 4 h (9 μmol m^-2^ s^-1^ UV-B and 2.92 μmol m^-2^ s^-1^ UV-A, Bio-Rad ChemiDoc^TM^ XRS UV-B lamps, catalog 1708097). The lamps used have emission spectra from 290 to 310 nm, and a peak at 302 nm. Samples were collected immediately after the light treatments from 10 to 14 h PM using fixtures mounted 30 cm above the plants. The bulbs were shielded with cellulose acetate filters (CA, 100 mm extra-clear cellulose acetate plastic, Tap Plastics, Mountain View, CA, United States); the CA filter excludes wavelengths lower than 290 nm without removing UV-B from longer wavelengths. This control was done in case some lower wavelength radiation was produced with lamps aging. A UV spectrum from a similar set up was previously shown in Casati and Walbot (2003). As a no UV-B control, plants were exposed for the same period of time under the same lamps covered with polyester filters that absorbs UV-B at wavelengths lower than 320 nm (PE, 100 mm clear polyester plastic; Tap Plastics). UV radiation was measured using a UV-B/UV-A radiometer (UV203 AB radiometer; Macam Photometrics). Samples were collected immediately after the light treatments.

For primary root elongation analysis, seedlings were grown in Petri dishes. Sterilized seeds were grown on MS growth medium and were kept in a vertical position in the growth chamber. Then, seedlings were UV-B irradiated for 1 h (9 μmol m^-2^ s^-1^) and then kept without UV-B for 3 days.

For flowering time analysis, plants were grown in the absence of UV-B for 9 days, and they were then irradiated with UV-B for 1 h a day until flowering, at an intensity of 9 μmol m^-2^ sec^-1^.

### Insertional T-DNA Mutants Identification

The identification of T-DNA insertion mutants was done using a PCR-based approach. Genomic DNA was isolated from leaves by a modified cetyl-trimetyl-ammonium bromide (CTAB) method (Sambrook and Russel, 2001) and PCR analysis was done using three combinations of primers. Two primers hybridize to specific genomic sequences (Supplementary Table S1) and one primer is located inside the left border of the T-DNA. The presence or absence of the T-DNA insertion in the genes allowed the identification of homozygous, heterozygous and WT plants.

### Quantitative RT-PCR

Total RNA purification and qRT-PCR was done as described in Fina and Casati (2015). Primers for each of the genes under study were designed using the PRIMER3 software (Rozen and Skaletsky, 2000) in order to amplify unique 150–250 bp products (Supplementary Table S1). Gene expressions were normalized to the *A. thaliana* calcium dependent protein kinase3 (*CPK3*, Supplementary Table S1). The expression of this gene has been previously shown not to be UV-B regulated in Arabidopsis (Ulm et al., 2004).

### DNA Damage Analysis

Cyclobutane pyrimidine dimers accumulation was quantified using an assay described in Stapleton et al. (1993). Monoclonal antibodies against CPDs (TDM-2) were from Cosmo Bio Co., Ltd. (Japan). UV-B treatments were performed both under light and dark conditions; plants irradiated under dark conditions were allowed to recover for 2 h under light or dark conditions. After the treatments, plant samples (0.1 g) were collected and immediately immersed in liquid nitrogen and stored at –80°C. 1.5 μg of the extracted DNA using a modified cetyl-trimetyl-ammonium bromide (CTAB) method was denatured in 0.3 M NaOH for 10 min. Samples were dot blotted onto a nylon membrane (Perkin Elmer life Sciences, Inc.) in sextuplicate. The blotted membrane was incubated for 2 h at 80°C and then blocked with a buffer containing 20 mM Tris-HCl, pH 7.6, 137 mM NaCl (TBS) and 5% (p/v) dried milk for 1 h at room temperature at 4°C. After this, the membrane was washed with TBS and incubated with TDM-2 antibodies (1:2000 in TBS) overnight at 4°C with agitation. Unbound antibody was washed away and secondary antibody conjugated to alkaline phosphatase (1:3000; BioRad) was added. The blot was washed several times and it was developed by the addition of NBT and BCIP. Quantification was done by densitometry of the dot blots using ImageQuant software version 5.2. DNA was quantified fluormetrically using the Qubit dsDNA assay kit (Invitrogen), and checked in a 1% (w/v) agarose gels after quantification.

### Root Length Measurements

Seedlings were grown in Petri dishes as described above. Then, seedlings were irradiated with UV-B for 1h (9 μmol m^-2^ sec^-1^) and then maintained in the absence of UV-B for 3 days. Plates were photographed before and after the treatment (1, 2, 3, and 4 days after), and the images were examined using the ImageJ program. Root lengths were measured by using a line traced along the root.

### Rosette Area Quantification

Approximately 20 seeds were sown per tray, in a spatial disposition to avoid superposition during plant growth. Twelve days after sowing (DAS), a group of plants were irradiated with a single UV-B treatment during 4 h at 9 μmol m^-2^ s^-1^; while a different group was maintained as control and were not UV-B irradiated. After the different treatments, the plants were kept in a growth chamber in the absence of UV-B. Photographs were taken every 3 days, and total leaf or rosette area of each plant was quantified using the ImageJ software.

### Microscopic Observations

Leaves were fixed using a solution containing 50% (v/v) ethanol; 5% (v/v) acetic acid, and 3,7% (v/v) formaldehyde; and then cleared with a solution of containing 200 g chloral hydrate, 20 g glycerol, and 50 ml dH_2_O, as described in Horiguchi et al. (2005). Leaf images were then acquired through a differential interference contrast (DIC) microscopy; and area was quantified using ImageJ image analysis software. Palisade leaf cells were observed by DIC microscopy, the area of palisade cells was determined, and leaf blade area was divided by this value to calculate the total number of palisade cells in the subepidermal layer. Twenty palisade cells were measured in each leaf in order to determine the cell area. Experiments were done in duplicate with at least 10 leaves with similar results.

### Flow Cytometric Analysis of Leaf #5

Ten leaves were cut with a razor blade in 1 ml of buffer (45 mM MgCl_2_, 30 mM sodium citrate, 20 mM 3-[N-morpholino]propane- sulfonic acid, pH 7.0, and 1% Triton X-100; Galbraith et al., 1991). The supernatants were filtered over a 30 μm mesh. Then, 1 μl of 4’,6-diamidino-2-phenylindole from a stock of 1 mg.ml^-1^ was added, supplemented with RNase at 50 mg.ml^-1^ and read through the Cell Sorter BD FACSAria II flow cytometer. The endoreduplication index (EI) was calculated from the percentage values of each ploidy class with the formula: EI = [(0 × %2C)+(1 × %4C)+(2 × %8C)+ (3 × %16C)+(4 × %32C)]/100 as described in Barrow and Meister (2003). This experiment was done in triplicate, each time using at least five plants corresponding to each treatment/genotype. In every experiment, for each treatment/genotype, at least 5,000 nuclei were analyzed.

### Flowering Time Analysis

For flowering time analysis, plants were grown in the absence of UV-B for 9 days, and then they were irradiated with UV-B for 1 h per day until flowering, at an intensity of 9 μmol m^-2^ s^-1^. For flowering phenotype determination, the days before flowering and the number of rosette leaves were counted at flowering for at least 12 individual plants.

### Chromatin Immunoprecipitation Experiments

For ChIP experiments, whole seedlings irradiated with UV-B for 1 day or kept under control conditions in the absence of UV-B were used. ChIP experiments were done out as previously described in Casati et al. (2008) using 4 μL of anti-acetylated H3 at the N-terminal domain (06-599, Upstate Biotechnology, Lake Placid, NY, United States) or anti-H3 (ab1791, Abcam, Cambridge, MA, United States) antibodies. Three biological replicates from each sample were used. ChIP experiments were quantified by qPCR in triplicates using primers in Supplementary Table S1. Data was normalized to values obtained using input DNA before immunoprecipitation.

### Statistical Analysis

Statistical analysis was done using ANOVA models (Tukey test) or alternatively Student’s *t*-test (Welch’s *T*-tests), using untransformed data.

## Results

### UV-B Regulation of HAF and HAC Genes and DNA Damage and Repair Analysis

Histone acetylation has been demonstrated to be important in UV-B responses in Arabidopsis (Campi et al., 2012; Fina and Casati, 2015; Falcone Ferreyra et al., 2016). Specifically, histone acetyltransferases from the MYST and the GNAT families participate in different UV-B responses. While UV-B induces the expression of both *HAM1* and *HAM2* (Campi et al., 2012); neither of the *HAG* transcripts are UV-B regulated (Fina and Casati, 2015). HAM1 and HAM2 proteins are required for efficiently repair of DNA damage after UV-B exposure, and *HAM1* and *HAM2* deficient plants show increased damage after exposure (Campi et al., 2012). In contrast, *HAG3* deficient plants accumulate lower cyclobutane dimers (CPDs) after UV-B irradiation (Fina and Casati, 2015).

**FIGURE 1:**
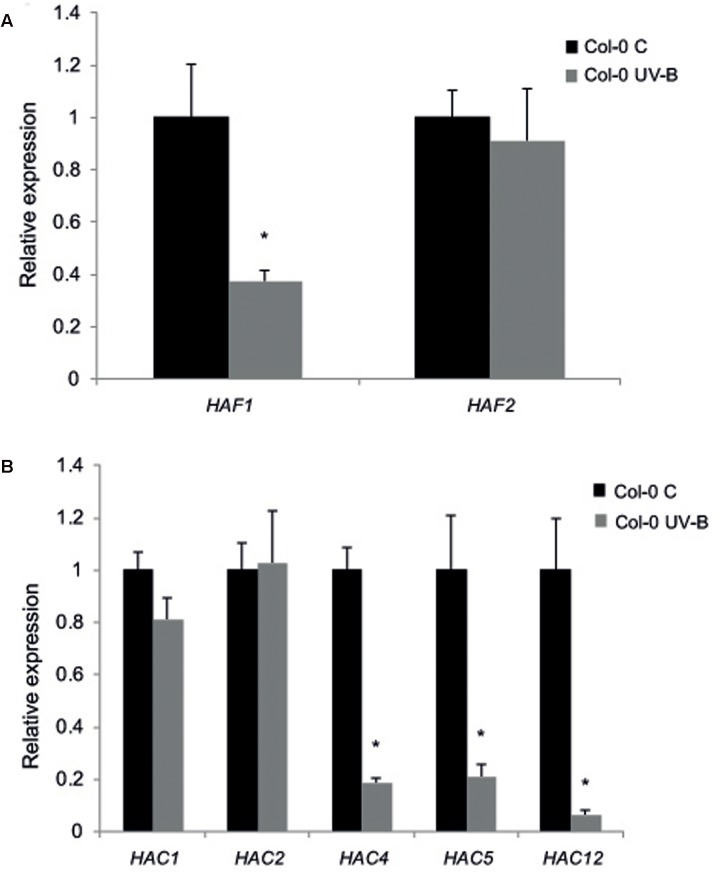
Relative expression of *HAF1* and *HAF2* **(A)** and *HAC1, HAC2, HAC4, HAC5* and *HAC12* **(B)** transcripts by RT-qPCR. Col-0 Arabidopsis plants were irradiated with UV-B for 4 h (UV-B) or were kept under control conditions without UV-B (control, C). Expression values are relative to the *CPK3* control. Data show mean values ±SEM of at least three independent experiments. Statistical significance was analyzed using Student’s *t*-test (Welch’s *T*-test) with *P* < 0.05; differences from the control are marked with an asterisk.

Thus, as a first attempt to examine the role of histone acetyltranferases from the HAF and the HAC families, we investigated if the HATs from these two families are UV-B regulated. WT plants of the Col-0 ecotype grown in the absence of UV-B for 4 weeks were irradiated with UV-B using lamps at an intensity of 9 μmol m^-2^ s^-1^ for 4 h in a growth chamber. The intensity used in the experiments is similar to solar UV-B at noon during summer time in Rosario, Argentina. Alternatively, plants were kept under control conditions in the absence of UV-B. Leaf tissue from plants under both conditions was collected for RNA extraction and qRT-PCR analysis. While *HAF1*, *HAC4*, *HAC5*, and *HAC12* were down-regulated by UV-B; *HAF2*, *HAC1*, and *HAC2* transcripts levels were not changed by the treatment (**Figure 1**).

To further explore the role of all these proteins in UV-B responses, *A. thaliana* lines defective in *HAF* and *HAC* transcripts were identified. For *HAF2*, *HAC1*, *HAC4*, *HAC5*, and *HAC12*, two T-DNA insertional lines with insertions in different parts of the genes were identified (Supplementary Figure S1) as described in Materials and Methods. The consequences of the insertion of the T-DNA in each gene were confirmed by RT-PCR on homozygous mutant plants, demonstrating that most mutations were knockout for the expression of each gene, except for *haf1-3* and *haf2-3*, which showed decreased transcript levels (Supplementary Figure S1). For *HAF1*, only one T-DNA insertional line was obtained (Supplementary Figure S1). Thus, for *HAF1* and also for *HAC2*, several independent RNAi lines were obtained from the Plant Chromatin collection (see Materials and methods). Both *haf1* and *hac2* RNAi transgenic lines had a significant reduction of *HAF1* and *HAC2* transcripts, respectively, in comparison to WT plants (Supplementary Figure S2).

**FIGURE 2:**
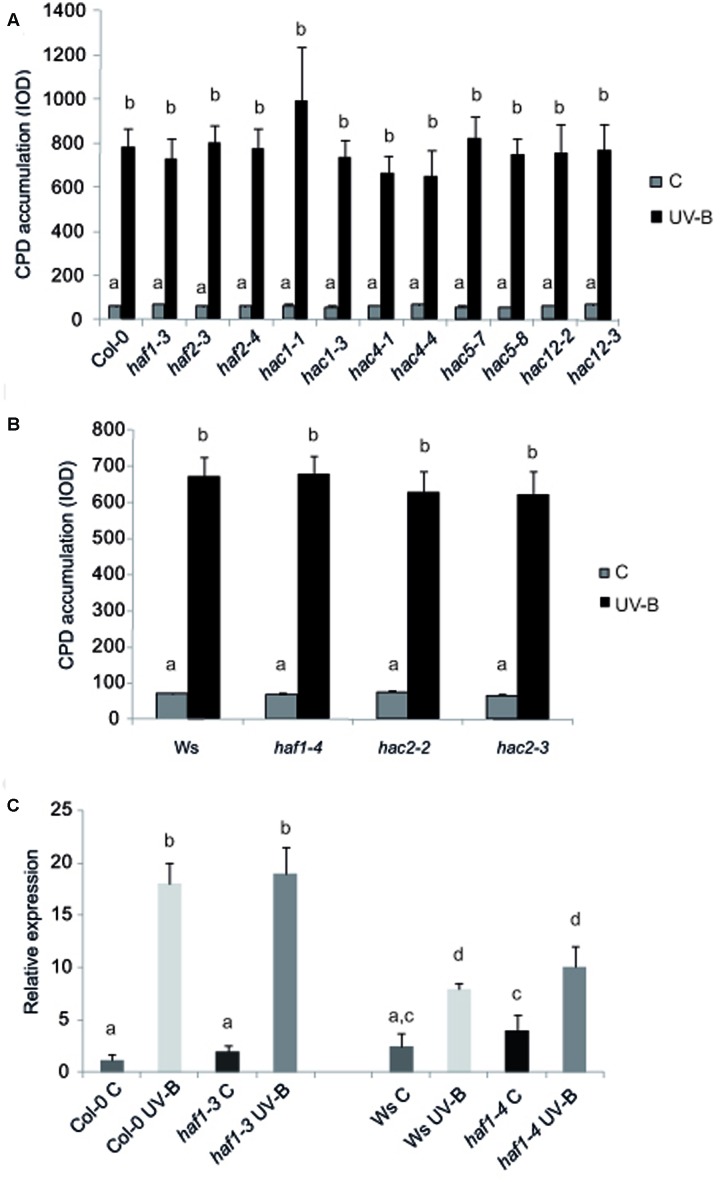
CPD levels in the DNA of WT Col-0, *haf* and *hac* mutants **(A)**, and WT Ws, *haf* and *hac* deficient plants **(B)** under control conditions in the absence of UV-B (control, C) and after UV-B exposure for 4h. Experiments were done under conditions that allowed photorepair in the light. 2 μg of DNA was loaded in each well. CPD levels are indicated as integrated optical density (IOD) values. Results represent the average ±SEM of six independent biological replicates. Statistical significance was analyzed using ANOVA, Tukey test with *P* < 0.05. **(C)** Relative expression of *UVR2* transcripts by qRT-PCR. WT Col-0 and Ws, and *haf1-3* and *haf1-4* mutant plants were irradiated with UV-B for 4 h or kept under control conditions without UV-B. Data show mean values 6 SD of at least three independent experiments. Different letters indicate significant statistical differences (*P* < 0.05).

To test if HAF and HAC proteins participate in UV-B induced DNA damage and repair, we grew *A. thaliana* WT plants and plants deficient in the expression of each *HAF* and *HAC* genes in the absence of UV-B for 4-weeks. Plants were then exposed to UV-B for 4 h. Different plants were exposed with the same lamps covered with a PE that absorbs UV-B as controls. DNA was extracted from samples collected immediately after the treatment for the analysis of CPD accumulation. Under control conditions in the absence of UV-B, the steady-state levels of CPDs in WT and all mutants were low and similar in all samples (**Figure 2**). Interestingly, UV-B caused a similar CPD accumulation in all lines, including WT plants (**Figure 2**). These results were comparable to those previously reported for *HAG1* and *HAG2* deficient plants and WT plants from the Col-0 and Ws lines (Campi et al., 2012; Lario et al., 2015; Fina and Casati, 2015); but differed to what was determined for *ham* and *hag3* mutants (Campi et al., 2012; Fina and Casati, 2015). Because it is possible that subtle effects of HAF or HAC activity on CPD removal might be masked by photolyase activity, the UV-B treatments were repeated in the absence of white light that allow photorepair. Samples were collected immediately after the treatments and also after 2 and 4 h of recovery in the absence of UV-B, both under light or dark conditions. For all plants, CPD accumulation was similar as that in WT plants under the same conditions. Results obtained with *haf1-3* and *hac1-3* plants are presented in Supplementary Figure S3. Supplementary Figure S3 shows that *haf1-3, hac1-3*, and Col-0 plants accumulate similar CPDs under the different experimental conditions, while *ham1-1* mutants, which previously showed increased DNA damage after UV-B exposure (Campi et al., 2012) had significant higher CPD levels, both in the presence or absence of white light.

### Growth Analysis of Plants with Decreased Levels of HAF and HAC Transcripts

**FIGURE 3:**
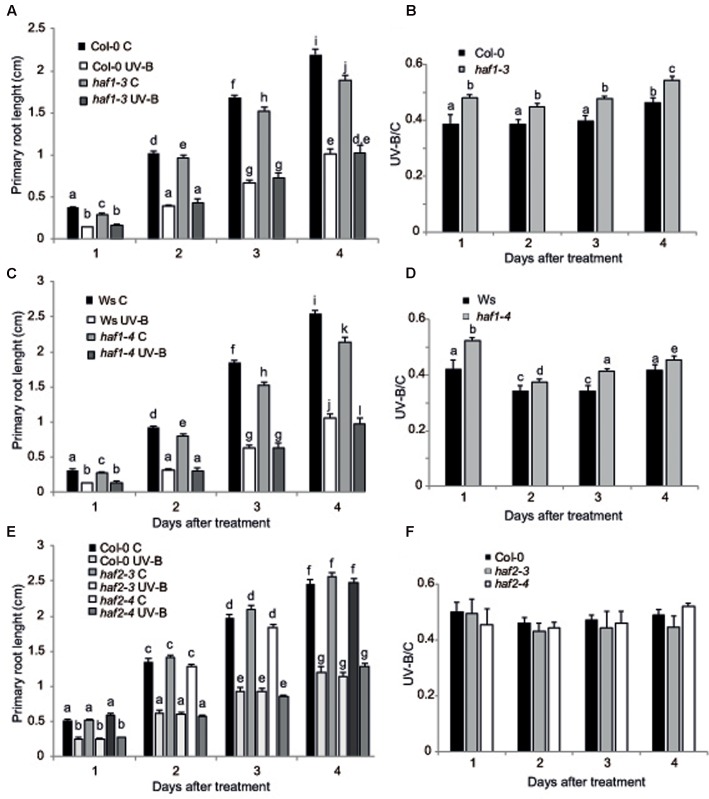
Primary root inhibition assays in WT, *haf1* and *haf2* plants after UV-B exposure. Graph of average root lengths in Col-0 and *haf1-3* plants **(A)**, Ws and *hag1-4* plants **(C)**, and Col and *haf2-3* and *haf2-4* plants **(E)** up to 4 days after a UV-B treatment or under control conditions in the absence of UV-B (control, C). Statistical significance was analyzed using ANOVA, Tukey test with *P* < 0.05; differences from the control are marked with different letters. The average root lengths after UV-B exposure relative to the length in control seedlings is shown in **(B)**, **(D,F)**; differences from the control are marked with different letters. The differences in primary root length of WT plants in the different panels are due to differences in developmental phases of the seedlings used when each UV-B irradiation was done in each experiment. Results represent the average of 20 biological replicates ±SEM.

UV-B radiation induces biomass reduction in plants depending on the intensity and dose of the exposure (Bornman and Teramura, 1993). Thus, UV-B sensitivity in the *haf* and *hac* lines was investigated by inhibition of primary root elongation assays (Tong et al., 2008). One day after the end of the UV-B treatment, all plants showed a significant decrease in primary root elongation in comparison to control non-irradiated plants (**Figure 3** and Supplementary Figure S4). Despite this, *haf1* plants showed a lower inhibition of primary root elongation than WT plants, this difference continued 4 days after the treatment (**Figure 3**). On the contrary, all *hac* and *haf2* plants analyzed showed a similar decrease by UV-B in primary root growth as WT plants (**Figure 3** and Supplementary Figures S4, S5). It is interesting to note that although the primary root length of both *hac1* and *hac4* seedlings was significantly different than that from WT plants under control conditions and after UV-B exposure (Supplementary Figures S4, S5), the UV-B/C ratio was similar as that in WT plants. Thus, although HAC1 and HAC4 may have a role in root development, this effect is independent of the UV-B treatment. Together, our results demonstrate that *haf1* plants show a lower sensitivity to root growth inhibition by UV-B than WT plants.

**FIGURE 4:**
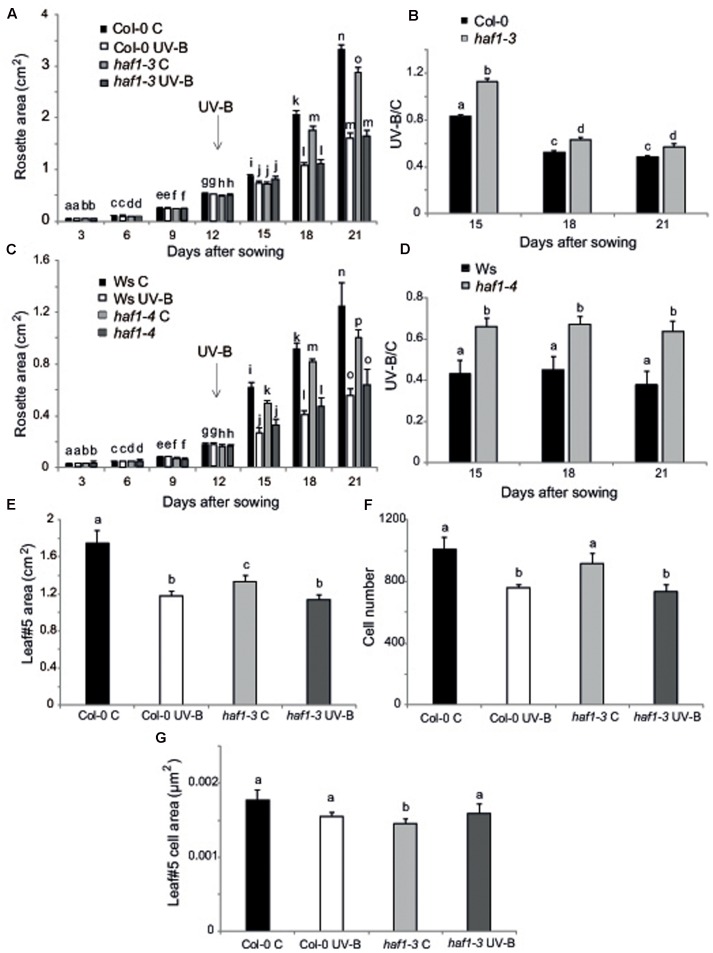
Plant growth inhibition by UV-B in *haf1* plants. WT (Ws and Col-0) and *haf1-3* and *haf1-4* plants were treated with UV-B radiation for 4 h (9 μmol m^-2^ s^-1^, right) or were kept under conditions in the absence of UV-B. **(A,C)** Rosette area of control and UV-B treated Col-0 and *haf1-3* **(A)**, and Ws and *haf1-4* **(C)** plants measured every 3 days from germination until 21 DAS. Plants were UV-B treated 12 DAS (indicated with an arrow). **(B,D)** The ratio of rosette areas of UV-B treated vs control plants for each line is shown. Results represent the average of 10 biological replicates ±SEM. Different letters denote statistical differences applying Student’s *t*-test (*P* < 0.05). **(E–G)** UV-B effect in leaf development in *haf1-3* plants. Relative average leaf area **(E)**, estimated cell number **(F)** and cell area **(G)** of fully expanded leaf #5 from UV-B treated versus control Col-0 and *haf1-3* Arabidopsis plants. Results represent the average of 10 biological replicates ±SEM. Statistical significance was analyzed using ANOVA, Tukey test with *P* < 0.05; differences from the control are marked with different letters.

On the other hand, inhibition of leaf growth is one of the most consistent plant responses to UV-B exposure, and this radiation has been demonstrated to limit leaf growth in different species (Ballaré et al., 2001; Flint et al., 2003; Radziejwoski et al., 2011; Casadevall et al., 2013). We have previously reported that UV-B radiation at an intensity of 9 μmol m^-2^ s^-1^ for 4 h decreased the rosette area in Arabidopsis plants; this decrease in rosette size is a consequence of a decrease in leaf area (Casadevall et al., 2013). In these plants, leaf #5 area, which was in active proliferation when plants were irradiated, was significantly reduced in UV-B irradiated plants because they have less cells, whereas cell area was similar in UV-B treated and control leaves, indicating that UV-B inhibits cell proliferation (Casadevall et al., 2013). Interestingly, we also showed that *hag3* RNAi plants had a significant lower inhibition of plant growth than WT plants (Fina and Casati, 2015). Thus, to analyze if plant growth inhibition by UV-B involves the participation of any or some HAF and/or HAC activities, we analyzed growth of Arabidopsis plants deficient in each HAF and HAC proteins that were grown in the absence of UV-B, and, 12 DAS, were either irradiated with UV-B for 4 h at 9 μmol m^-2^ sec^-1^ or were kept under control conditions. Plants were then allowed to grow in the absence of UV-B and rosette area was measured every 3 days up to 21 DAS. Under control conditions, all plants except *HAF1* deficient plants had a similar rosette area as WT plants; while *haf1* plants were smaller than WT both in the Ws or Col 0 backgrounds (**Figure 4A** and Supplementary Figures S4, S5), suggesting that HAF1 may have a role in plant development. After a single UV-B treatment for 4 h, all plants showed a decrease in the rosette area; however, only *haf1* plants had a significant lower inhibition of plant growth than WT plants; with *HAC*s and *HAF2* deficient plants showing a similar decrease in rosette area as WT plants (**Figures 4A–D** and Supplementary Figures S4, S5). In this way, after the UV-B treatment, *haf1* and WT plants showed a similar rosette area (**Figures 4A,C** and Supplementary Figures S5, S6).

To further investigate the role of HAF1 in the inhibition of plant growth by UV-B, we analyzed and compared the effect of this radiation in leaf #5 growth, which was proliferating at the moment of the UV-B treatment, from *haf1* and WT plants. In *haf1* control plants that were not exposed to UV-B, leaf #5 was smaller than the same leaf in WT plants; however, in UV-B exposed plants, although the average leaf #5 area in *haf1* plants was reduced by the treatment, it was similar to that of WT plants after exposure (**Figure 4E** and Supplementary Figure S6). Similarly, as also previously determined in leaves from *hag3* mutants (Fina and Casati, 2015), in control plants, *haf1* leaf #5 was smaller because it had cells with reduced size, while the number of cells in leaf #5 from both plants was similar (**Figures 4F,G**). Despite this, after UV-B exposure, *haf1* and WT plants had a similar inhibition of cell proliferation (**Figure 4F**), but *haf1* plants presented an increase in cell area not measured in WT leaf #5 cells; and as a consequence, cell area in *haf1* and WT leaf #5 after UV-B exposure were similar (**Figure 4G**).

**FIGURE 5:**
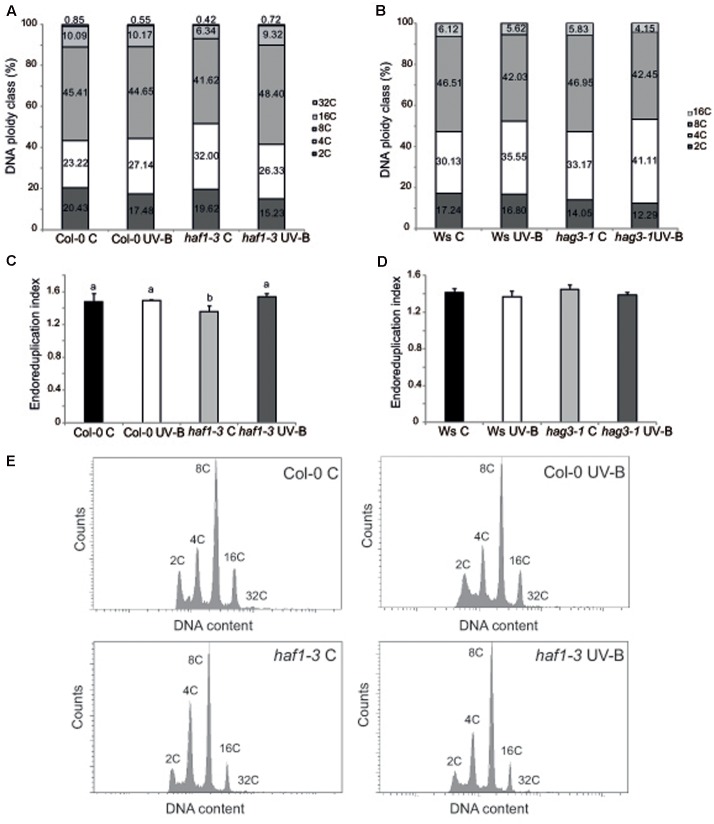
Analysis of DNA ploidy in leaf #5 of Col-0 and *haf1-3* plants. Comparison of the nuclear DNA content of Col-0 and *haf1-3* **(A)**, and Ws and *hag3-1* **(B)** leaf #5 from UV-B treated and control Arabidopsis plants determined by flow cytometry. **(C,D)** Endoreduplication index (EI) of cells in leaf #5 from UV-B treated and control Col-0 and *haf1-3* **(C)**, and Ws and *hag3-1* **(D)** Arabidopsis plants. Statistical significance was analyzed using ANOVA, Tukey test with *P* < 0.05; differences from the control are marked with different letters. **(E)** Flow cytometry profiles of the nuclear DNA content of Col-0 and *haf1-3* leaf #5 from UV-B treated and control Arabidopsis plants.

Cell size can be in part a consequence of its DNA content (Melaragno et al., 1993; Sugimoto-Shirasu and Roberts, 2003). Thus, to investigate if differences in cell area in *haf1* leaf #5 were due to changes in DNA ploidy, leaf #5 ploidy levels were measured through flow cytometry after UV-B exposure or in control conditions in the absence of UV-B. **Figure 5** shows that, in Col-0 plants, the endoreduplication index (EI) was similar in leaf #5 under control conditions and after UV-B exposure. Interestingly, in *haf1* leaves under control conditions, the EI was lower than that of Col-0 plants, but reached WT values after UV-B exposure (**Figure 5C**). These changes in EI correlate with measurements of cell area presented in **Figure 4G**. As observed in *haf1* leaf #5 under control conditions, there is a strong decrease in the relative amount of cells with 8C and 16C DNA content in comparison to WT control leaves (**Figure 5A**). In contrast, endoreduplication was stimulated in the *haf1* leaf #5 after UV-B exposure, as exemplified by the increase in the 8C and 16C population. For both lines, there was a strong correlation between the DNA content of one cell and its average size, with the 8C and 16C cells accounting mostly for the differences observed in cell area (**Figures 4**, **5**). Together, our data indicate that the deficiency of HAF1 expression under control conditions decreases endoreduplication and therefore cell area, but after UV-B exposure ploidy levels are regained with an increase in cell area, possibly as a compensation effect. In this way, leaf #5 from *haf1* plants, which have a cell proliferation defect under UV-B, can reach a final leaf size without being further reduced after exposure.

Previously, we also demonstrated that in *hag3* plants in the absence of UV-B, leaf #5 was also smaller than WT leaf #5 because it had smaller cells; but in UV-B exposed plants, average leaf #5 area in *hag3* plants was similar to that of WT plants and they had similar cell areas (Fina and Casati, 2015). To analyze if this phenotype in *hag3* plants was also a consequence of changes in the ploidy levels by UV-B as observed for *haf1* plants; we therefore analyzed leaf #5 ploidy levels in these plants through flow cytometry. As shown in **Figures 5B,D** EI and ploidy levels in the DNA were similar in *hag3* and WT plants in the Ws background, both under control conditions and after UV-B exposure. Thus, changes in cell area due to deficiencies in HAF1 and HAG3 activities are probably mediated by different mechanisms.

### HAF1 Role in Endoreduplication Could Be Mediated by E2F Transcription Factors

**FIGURE 6:**
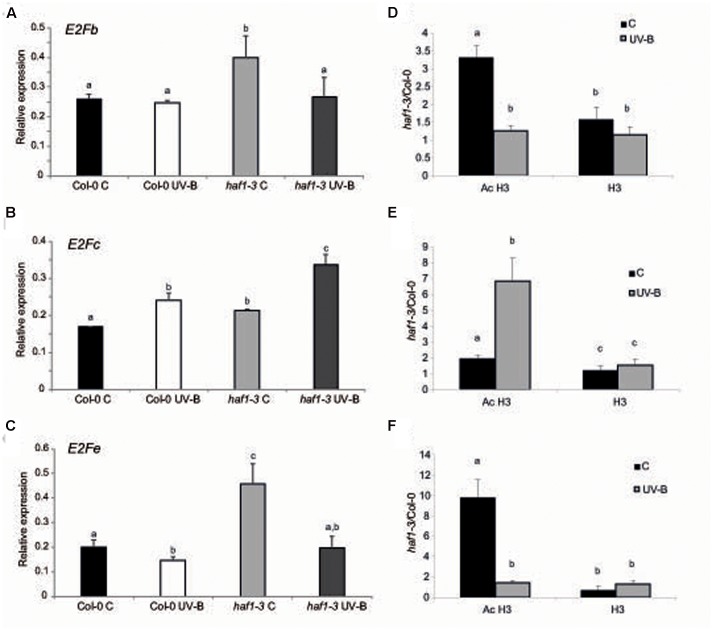
Relative expression of *E2Fb* **(A)**, *E2Fc* **(B)**, and *E2Fe* **(C)** transcripts in Col-0 and *haf1-3* plants by RT-qPCR and AcH3 status of the genes **(D–F).** Plants were irradiated with UV-B for 4 h (UV-B) or were kept under control conditions without UV-B (control, C). Expression values are relative to the *CPK3* control. Data show mean values ±SEM of at least three independent experiments. Statistical significance was analyzed using ANOVA, Tukey test with *P* < 0.05; differences from the control are marked with different letters. **(D–F)** ChIP-qPCR analysis of Ac H3 and total H3 associated to the region 1 of *E2Fb* **(D)**, *E2Fc* **(E)** and *E2Fe* **(F)** depicted in Supplementary Figure S7 in UV-B irradiated and control Col-0 and *haf1-3* plants. Data was normalized to values obtained using input DNA before immunoprecipitation. Results represent the average of 3 biological replicates ±SEM. Statistical significance was analyzed using ANOVA, Tukey test with *P* < 0.05; differences from the control are marked with different letters.

It was previously demonstrated that the change from a mitotic cell cycle into an endoreduplication cycle is controlled by the E2Fe/DEL1 transcription factor, which represses the endocycle onset (Vlieghe et al., 2005; Lammens et al., 2008). Moreover, *E2Fe* is transcriptionally regulated by the classical E2Fb and E2Fc transcription factors; these two proteins antagonistically control *E2Fe* transcript levels through the competition for a single E2F cis-acting binding site (Berckmans et al., 2011). While E2Fb activates transcription of *E2Fe*, E2Fc is a repressor of this gene. Thus, we analyzed if *E2Fb*, *E2Fc* and *E2Fe* transcript levels were altered in *HAF1* deficient plants. While *E2Fe* transcripts were significantly higher in *haf1* plants under control conditions than in WT plants, after UV-B exposure, levels decreased about 2.3-fold in *HAF1* deficient plants, reaching similar levels as those in WT values (**Figure 6C**). *E2Fe* expression levels directly correlated with *E2Fb* levels in the WT and the mutant lines under the two light conditions studied (**Figure 6A**), and inversely correlated with those of *E2Fc* (**Figure 6C**), as previously reported (Berckmans et al., 2011). In this way, our results suggest that HAF1, directly or indirectly, regulate *E2Fb*, *E2Fc*, and *E2Fe* transcript levels, possibly by acetylating histones associated with these genes, or alternatively with regulators of their expression.

As shown in Supplementary Figure S7, all three *E2Fb*, *E2Fc*, and *E2Fe* genes show high histone acetylation in the chromatin associated to their 5′ regions (Sequeira-Mendes et al., 2014); thus, direct or indirect changes in histone acetylation in the 5′ regions of these genes by HAF1 under UV-B conditions resulting in changes in expression levels of these transcription factors may be at least one cause of the differences in DNA ploidy levels measured. As shown in Supplementary Figure S7, the 5′ end of *E2Fb*, *E2Fc*, and *E2Fe* genes associates to acetylated H3 at the N-terminal domain; therefore, to further explore the hypothesis that acetylated H3 contribute to the transcriptional response of E2F transcription factors to UV-B in WT Col-0 and *HAF1* deficient plants, ChIP analysis was performed using commercially available antibodies specific for acetylated Lys residues in the N-terminal tail of histone H3. As a control, antibodies against total H3 histone were used. DNA recovered after immune precipitation was screened via quantitative PCR for the presence of 5′ regions of *E2Fb*, *E2Fc*, and *E2Fe* genes. To evaluate nonspecific binding, the quantitative PCR was also done with samples incubated in the absence of antibody; all ChIP samples were normalized to total input DNA from sonicated nuclei to evaluate the selective recovery of the gene segments. 5′ regions of *E2Fb* and *E2Fe* were enriched significantly in the fractions immunoprecipitated with anti-acetylated H3 in control samples from the *haf1-3* mutant relative to the Col-0 control; however, similar immunoprecipitated DNA levels were measured in UV-B–irradiated samples from both genotypes (**Figures 6D,F**). Thus, higher H3 acetylation under control conditions in the absence of UV-B of *E2Fb* and *E2Fe* 5′ regions is correlated with an increase in transcript abundance. On the other hand, higher immunoprecipitated DNA levels using anti-acetylated H3 antibodies were observed in *E2Fc* 5’regions from *haf1-3* mutants than in Col-0 plants, both under control conditions and after UV-B exposure; despite this, enrichment was significantly higher after UV-B exposure (**Figure 6E**). Again, the increase in acetylation of H3 associated to *E2Fc* in *HAF1* deficient plants correlates with higher transcript levels. Similar immunoprecipitated DNA levels were observed in control and UV-B–irradiated samples from all genotypes when H3 antibodies were was for immunoprecipitation (**Figures 6D,E**). Collectively, the ChIP analyses indicate that differences in H3 acetylation exist in the chromatin associated with *E2Fb*, *E2Fc* and *E2Fe* transcription factors in WT Col-0 plants and in the *haf1-3* mutant under control conditions in the absence of UV-B and after exposure, and these changes correlate with differences in transcript abundance.

### HAC1 Has a Role in the Regulation of Flowering Time under UV-B Conditions

The results presented in this manuscript show that histone acetyltransferases from the HAC family do not participate in DNA repair after UV-B exposure, or in growth changes elicited by this radiation. As mentioned in the Introduction, HAC1, HAC5, and HAC12 promote flowering regulating transcription of *FLOWERING LOCUS C* (*FLC*), a major floral repressor (Deng et al., 2007; Han et al., 2006). Interestingly, *hac1* single mutants have a late-flowering phenotype, while *hac5* and *hac12* single mutants do not show any visible flowering phenotype (Han et al., 2006). Thus, we analyzed if plants deficient in these proteins have affected flowering time after UV-B exposure. WT Col-0 plants and *hac1*, *hac5* and *hac12* mutants in the same genetic background were grown in the absence of UV-B for 9 days, and they were then irradiated with UV-B for 1 h per day until flowering, at an intensity of 9 μmol m^-2^ s^-1^. Flowering time was analyzed by both measuring the number of rosette leaves formed before flowering, and by counting the days from germination till flowering.

**FIGURE 7:**
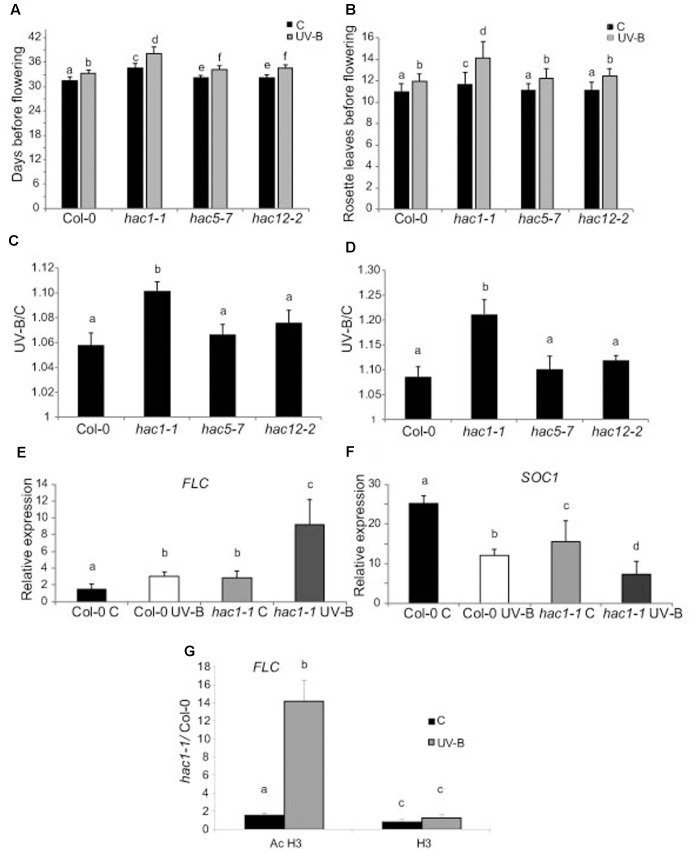
UV-B effect in flowering time in WT, *hac1*, *hac5*, and *hac12* mutants. Days before flowering **(A)** and number of leaves at flowering time **(B)** for WT, *hac1-1*, *hac5-7*, and *hac12-2* mutants under control conditions, or in plants that were grown in the absence of UV-B for 9 days, and they were then irradiated with UV-B for 1 h/day until flowering, at an intensity of 9 μmol m^-2^ s^-1^ under long day photoperiod. **(C,D)** The ratio of flowering time, analyzed by counting the days before flowering **(C)** and the number of leaves at flowering time **(D)** of UV-B treated vs control plants for each line is shown. Results represent the average of 10 biological replicates ±SEM. **(E,F)** Relative expression of *FLC* **(E)** and *SOC1* **(F)** transcripts in Col-0 and *hac1-1* plants by RT-qPCR. Plants were irradiated with UV-B for 4 h (UV-B) or were kept under control conditions without UV-B **(C)**. Expression values are relative to the *CPK3* control. Data show mean values ±SEM of at least three independent experiments. Statistical significance was analyzed using ANOVA, Tukey test with *P* < 0.05; differences from the control are marked with different letters. **(G)** ChIP-qPCR analysis of Ac H3 and total H3 associated to the promoter region of *FLC* in UV-B irradiated and control Col-0 and *hac1-1* plants. Data was normalized to values obtained using input DNA before immunoprecipitation Results represent the average of three biological replicates ±SEM. Statistical significance was analyzed using ANOVA, Tukey test with *P* < 0.05; differences from the control are marked with different letters.

**Figures 7A,B** show that flowering time was delayed in *hac1* mutants, as previously reported (Han et al., 2006; Deng et al., 2007). Interestingly, flowering time was also delayed in all UV-B irradiated plants, but this delay was more important in *hac1* plants than in Col-0, *hac5* and *hac12* plants (**Figures 7A,D**). It was previously reported that the late-flowering phenotype of *hac1* mutants was mediated by the flowering repressor FLOWERING LOCUS C (FLC), which transcript levels are increased in *hac1* mutants (Han et al., 2006; Deng et al., 2007). This negative regulation of *FLC* mediated by HAC1 probably involves reversible protein acetylation (Han et al., 2006; Deng et al., 2007). Thus, we analyzed *FLC* levels in *hac1* and WT plants irradiated with UV-B. As shown in **Figure 7E**, *FLC* levels were higher in *hac1* mutants than in WT plants, both under control conditions and after UV-B exposure. Interestingly, in both genotypes, *FLC* was induced by UV-B, but levels after exposure in *hac1* plants were significantly higher than in WT (about threefold higher in *hac1* than in WT plants). FLC is a repressor of *SUPPRESSOR of OVEREXPRESSION of CONSTANS 1* (*SOC1*), which encodes a MADS box transcription factor and integrates multiple flowering signals. *SOC1* was also differentially and inversely regulated in *hac1* plants and by UV-B radiation (**Figure 7F**). While levels in WT Col-0 plants were higher than in *hac1* plants, transcripts were also repressed by UV-B, showing 1.6-fold lower levels in *hac1* than in WT plants that were UV-B irradiated. In this way, *FLC* and *SOC1* expression correlates with the delay in flowering time observed in *hac1* mutants, and may mediate the delay in flowering time in *hac1* mutants after UV-B exposure.

To analyze if changes in the regulation of *FLC* expression in *HAC1* deficient plants by UV-B involves reversible histone acetylation, we analyzed if acetylated H3 contributed to the transcriptional response observed by ChIP analysis. Similar immunoprecipitated DNA levels were measured in control samples from both Col-0 and *hac1-1* plants; however, the 5′ region of *FLC* was significantly enriched in the fraction immunoprecipitated with anti-acetylated H3 in UV-B irradiated samples from the *hac1-1* mutant (**Figure 7G**). Thus, higher H3 acetylation after UV-B exposure of *FLC* 5’region is correlated with an increase in transcript abundance. Similar immunoprecipitated DNA levels were observed in control and UV-B–irradiated samples from both genotypes when H3 antibodies were used for immunoprecipitation (**Figure 7G**). In this way, the ChIP analyses demonstrate that differences in H3 acetylation exist in the chromatin associated with *FLC* in Col-0 and in the *hac1-1* mutant after UV-B exposure, and these changes parallel changes in transcript abundance.

## Discussion

In previous experiments, we showed that histone H3 and H4 acetylation is increased by UV-B radiation in maize and Arabidopsis plants; and plants treated with an inhibitor of histone acetylases showed increased DNA damage by UV-B (Casati et al., 2008; Campi et al., 2012). We also demonstrated that histone acetyltransferases from the HAG and HAM families participate in different responses of Arabidopsis plants exposed to UV-B radiation. While HAM1 and HAM2 are required for an efficient repair of UV-B damaged DNA (Campi et al., 2012); HAG3, probably as a subunit of the Elongator complex, negatively regulates transcription of UV-B regulated genes in Arabidopsis (Fina and Casati, 2015). In this work, to further investigate the link between UV-B and histone acetylation, we have examined the role of HATs from the HAC and HAF families in the same and different UV-B responses.

The results presented here demonstrate that neither HAC nor HAF acetyltrasferases participate in DNA damage and repair after UV-B exposure in Arabidopsis plants (**Figure 2**). Previously, Waterworth et al. (2015) demonstrated that the *haf1-3* mutant, which lacks the C-terminal bromodomain and is the same mutant used in the experiments presented here, showed hypersensitivity to X-rays and mitomycin C, suggesting that HAF1 participates in DNA repair processes. However, they also showed that it did not participate in other forms of abiotic stress responses, including treatment with methyl methanesulfonate, which causes alkylation to DNA bases. UV-B radiation principally produces covalent bonds between adjacent pyrimidines, giving rise to CPDs and 6-4 photoproducts, which are mostly repaired by photolyases under light conditions in plants (Friedberg et al., 1995). CPDs are the primary UV-B-induced DNA lesions accounting approximately 75% of UV-B-mediated total DNA damage, while pyrimidine (6–4) pyrimidone dimers are secondary lesions, whereas the minor damages include oxidized or hydrated bases, which as a consequence could give raise to strand breaks, and other breaks (for a revision see Manova and Gruszka, 2015). We have previously demonstrated that the UV-B levels used in our experiments produce some oxidative damage in the cells which may induce oxidative damage in the DNA (Falcone Ferreyra et al., 2016). Despite this, this oxidative damage did not induce any detectable oxidative damage in the DNA that could eventually generate strand breaks (Qüesta et al., 2013). It is interesting to note that only very high UV-B levels have been reported to induce oxidative damage in DNA (see Britt, 1996); while UV-B levels used in our study are similar to those in natural sunlight (Casati and Walbot, 2003). We showed that our UV-B treatment has no effect on the rates of somatic homologous or homeologous recombination, demonstrating that no strand break occurs after exposure (Lario et al., 2015). On the contrary, mitomycin C is a DNA cross-linking agent which damage is mostly repaired by homologous recombination (HR; Bleuyard et al., 2005). On the other hand, X-rays provoke different lesions through direct ionization of DNA producing strand breaks, or indirectly through an initial interaction with water, which results in the generation of reactive oxygen species and oxidative damage in DNA; both types of damage are mostly repaired by excision repair mechanisms (Manova and Gruszka, 2015). Because the repair machineries of HR and excision repair systems are both composed by several proteins, it is possible that they may be more influenced by chromatin organization and histone acetylation by HAF1 than photorepair by photolyase, which is comprised by only one polypeptide. Alternately, HAF1 may regulate the expression of certain DNA repair genes but not others. In this respect, qRT-PCR analysis of the photolyase *UVR2*, that encodes an enzyme that specifically repairs CPDs in Arabidopsis, showed that WT and *HAF1* deficient lines in different genetic backgrounds had similar and low expression levels under control conditions. Transcripts are increased after UV-B exposure and correlate with CPD accumulation (**Figure 2C**), suggesting that photorepair is not altered in *HAF1* deficient lines. Finally, we cannot rule out that redundancy may exist, so the role of one histone acetyltransferase in DNA damage and repair by UV-B may be masked by the activity of a different enzyme. However, because available data shows that HAC1 is predominant versus the other HACs, and because HAF1 and HAF2 are expressed during different stages of plant development; if any of the proteins characterized in this manuscript had a role in UV-B induced DNA damage, some differences in CPD accumulation should be observed.

In spite of this, we here provided evidence that HAF1 has a role in the inhibition of both leaf and root growth by UV-B. According to our results, this growth inhibition seems to be independent of DNA damage. Different works have reported a UV-B inhibition of hypocotyl and/or leaf growth through the activation of the UVR8 photoreceptor pathway, this decrease in plant growth is independent of DNA damage (Hectors et al., 2007, 2010; Favory et al., 2009; Gruber et al., 2010; Huang et al., 2012); moreover, the inhibition of root elongation by UV-B seems to be mediated by the RUS1 and RUS2 pathway (Tong et al., 2008; Leasure et al., 2009). This is also true for the UV-B leaf inhibition response observed in maize, in this species we demonstrated that UV-B levels present in solar radiation inhibits maize leaf growth without causing any accumulation of DNA damage (Fina et al., 2017). Despite *HAF1* is not a target of the UVR8 pathway under UV-B conditions (Brown et al., 2005; Favory et al., 2009), HAF1 probably participates in other UV-B regulated pathways, independently of UVR8 and DNA damage. Leaf #5 from *haf1* mutants in the absence of UV-B is smaller than the same leaf in WT plants; this is because *haf1* leaf #5 has smaller cells, while the number of cells is similar in leaf #5 from both plants (**Figure 4**). *haf1* leaves show a lower EI than WT leaves (**Figure 5**), suggesting that the decreased cell size in *haf1* leaves could be partly determined by DNA ploidy (Melaragno et al., 1993; Sugimoto-Shirasu and Roberts, 2003). Despite this, in UV-B exposed plants, leaf #5 area in *haf1* plants is similar to that of WT plants, with similar average cell area and cell number, showing a similar inhibition of cell proliferation (**Figure 4**). The increase in cell area in *haf1* leaves by UV-B, correlating with an increase in the EI, again suggests that cell size in *haf1* leaf #5 can be determined by its DNA content. It is important to highlight that in our previous work using plants deficient in the expression of different histone acetyltranferases from the HAG familiy (Fina and Casati, 2015) and also now in this work, we have also identified different histone acetyltransferases mutants that, although they have either a smaller rosette area or shorter roots than WT plants under control conditions, they also show an even smaller area or shorter roots after UV-B exposure, showing a UV-B/C ratio similar to that of WT plants, that have bigger rosettes and longer primary roots under both conditions. This is the case for example of *hag1* and *hag2* deficient plants reported in Fina and Casati (2015), these plants have a smaller rosette area than that of WT plants, both under control conditions and after UV-B exposure, showing a UV-B vs C ratio similar to that of WT plants; and of *hac1-1* and *hac2* deficient plants in this work, these plants have a shorter primary root than WT plants, both under control conditions and after UV-B exposure, with a UV-B vs C ratio similar to that of WT plants. Although *haf1* deficient plants are also smaller and have shorter roots, they are also less responsive than WT plants to the growth inhibition effect of UV-B radiation. Collectively, our data indicate that a deficiency in HAF1 under control conditions decreases endoreduplication and therefore cell area, but after UV-B exposure, ploidy levels are regained with an increase in cell area, possibly as a compensation effect. In this way, leaf #5 from *haf1* plants, which have a cell proliferation defect under UV-B, can reach a final leaf size and it is not further reduced after exposure. Moreover, endoreduplication has been previously suggested to assist in the protection against UV-B radiation, as it was demonstrated by the increased UV-B tolerance of the *uvi4* mutant that displays an increased DNA ploidy level (Hase et al., 2006). The compensation effect, where a deficiency of cell proliferation triggers cell expansion, has been reported several times and was also observed for *hag3* mutants exposed to UV-B (Horiguchi and Tsukaya, 2011; Fina and Casati, 2015). However, our data shows that in *hag3* mutants, the compensation effect does not depend on changes in DNA ploidy levels, as observed in *haf1* mutants. Additional experiments, for example the analysis of leaf growth in *HAF1* overexpressor lines after UV-B exposure, would provide better confirmations on the role of this protein in the regulation of plant growth under UV-B.

The atypical E2F transcription factor E2Fe/DEL1 is a central controller of the endocycle onset (Vlieghe et al., 2005; Lammens et al., 2008). In addition, E2Fe was identified as a transcriptional repressor of the CPD photolyase PHR1 in Arabidopsis (Radziejwoski et al., 2011). *E2Fe* is transcriptionally regulated by E2Fb and E2Fc; which antagonistically control *E2Fe* mRNA levels through the competition for a single E2F binding site (Berckmans et al., 2011). E2Fb activates transcription of *E2Fe*, while E2Fc is a repressor of this gene. In leaf #5 of *HAF1* deficient plants, *E2Fe* is higher expressed under control conditions than in WT plants; and after UV-B irradiation, levels decreased about 2.3-fold in *HAF1* deficient plants, showing similar expression levels as those in WT values (**Figure 6**). We found that *E2Fe* expression levels directly correlated with *E2Fb* levels in WT and *haf1* plants under the two light conditions studied, and inversely correlated with those of *E2Fc*, in agreement with previous reports (Berckmans et al., 2011). Thus, our results suggest that HAF1, directly or indirectly, could regulate *E2Fb*, *E2Fc* and *E2Fe* expression, possibly by acetylating histones associated with these genes, or alternatively with regulators of their expression. Interestingly, Sequeira-Mendes et al. (2014) demonstrated that the 5′regions of *E2Fb*, *E2Fc*, and *E2Fe* genes are associated to H4K5ac; H3K14ac; H3K9ac, which are typical marks of transcription start sites of transcribed regions (Supplementary Figure S7). Although the specificity of HAF1 activity on different histone residues is not known, direct or indirect changes in H3 and H4 acetylation of these residues in the 5′regions of these genes by HAF1 under UV-B conditions resulting in changes in expression levels of these transcription factors may be at least one cause of the differences in DNA ploidy levels measured in *haf1* plants. In fact, our results demonstrate that differences in H3 acetylation exist in the chromatin associated with *E2Fb*, *E2Fc*, and *E2Fe* transcription factors in Col-0 plants and in the *haf1-3* mutant under control conditions and after UV-B exposure (**Figure 6**), and these differences correlate with differences in transcript abundance. Thus, the *haf1-3* mutant fails directly or indirectly to introduce proper H3 acetylation on *E2F* genes, affecting in this way their expression and as a consequence DNA plody. Consistent with our results, Vlieghe et al. (2005) demonstrated that leaves of DEL1^OE^ transgenic plants showed a decreased area compared to that of WT plants. These plants had smaller cells; and this decrease in cell size was accompanied by lower levels of endoreduplication, suggesting that high expression of *E2Fe*/*DEL1* inhibits the mitotic cycle. Together, the urge for cell expansion could be the cause for the increased endoreduplication regulated by E2Fe measured after UV-B in *haf1* leaves.

On the other hand, we here demonstrated that *HAC1* deficient plant have a later flowering time after UV-B exposure than WT plants (**Figure 7**), this effect in flowering time is also independent of a DNA damage response. *HAC1* is not a regulated by the UVR8 pathway under UV-B conditions (Brown et al., 2005; Favory et al., 2009); therefore, and similarly as HAF1, HAC1 may participate in other UV-B regulated pathways, independently of UVR8 and DNA damage. In Arabidopsis, HAC1 deficiency causes pleiotropic developmental defects, such as a delay in flowering time, a shortened primary root, and decreased fertility (Han et al., 2006; Deng et al., 2007). In *hac1* mutants, transcript levels of the flowering repressor *FLC* were increased, indicating that the late-flowering phenotype of these mutants could be through the activity of FLC (Deng et al., 2007). Interestingly, histone modifications of FLC chromatin were not modified in *hac1* mutants, suggesting that HAC1 affects flowering by epigenetic modification of factors upstream of FLC (Deng et al., 2007). However, our results demonstrate that differences in H3 acetylation exist in the chromatin associated with *FLC* in Col-0 and in the *hac1-1* mutant after UV-B exposure, and these changes parallel changes in transcript abundance, suggesting that the *hac1-1* mutation affects the correct H3 acetylation on *FLC* only after UV-B exposure (**Figure 7G**). Our results show that *FLC* levels are higher in *hac1* mutants than in WT plants, both in the absence and after UV-B exposure. In both genotypes, *FLC* is induced by UV-B, but transcript levels in *hac1* plants after exposure are significantly higher than in WT plants. FLC is a repressor of SOC1, which integrates multiple flowering signals. *SOC1* is also differentially and inversely regulated in *hac1* and by UV-B radiation. Thus, *FLC* and *SOC1* expression through changes of H3 acetylation on the *FLC* gene correlates with the delay in flowering time observed in *hac1* mutants. Although we did not detect differences in flowering time by UV-B among the WT, *hac5* or *hac12* single mutants (**Figures 7C,D**), the conserved domain organizations and high sequence similarities of HAC1, HAC5 and HAC12 suggest that functional redundancy among these genes exist. In fact, previous experiments showed that although no delay in flowering time was measured in *hac5* or *hac12* single mutants, double mutants between the three genes (*hac1hac5*, *hac1hac12* and *hac5 hac12*) showed dramatically delayed flowering compared to that in WT or the *hac1* single mutant (Han et al., 2006). Thus, it is possible that HAC5 and/or HAC12 may also participate in the regulation of flowering time under UV-B conditions, but their activity could be replaced by other of the redundant family members.

In summary, the results presented in this manuscript, together with previous data, demonstrate that histone acetyltransferases have differential and active roles during UV-B exposure in Arabidopsis. While HAM family HATs are required for an efficient repair of UV-B damaged DNA; HAG3, HAF1 and HAC1 regulate the expression of genes in UV-B responses in Arabidopsis through changes in histone acetylation, which are required for developmental responses to this radiation. Despite this, we cannot rule out the participation of other HATs in different aspects of UV-B responses in Arabidopsis.

## Author Contributions

JF and PC designed the experiments. PC wrote the paper. JF, FM, SR, and FC performed the experiments.

## Conflict of Interest Statement

The authors declare that the research was conducted in the absence of any commercial or financial relationships that could be construed as a potential conflict of interest.

## Footnotes

1 http://www.chromdb.org/rnai/rnai_lines_info.html
