# Supplementary material for: HAC1 and HAF1 Histone Acetyltransferases Have Different Roles in UV-B Responses in Arabidopsis
Source: Front Plant Sci. 2017 Jul 10;8:1179. doi: 10.3389/fpls.2017.01179 (PMC5502275; doi:10.3389/fpls.2017.01179)
Supplement: Supplementary file 1 [file Table_1.PDF]

Table S1. Primers used in this study

Primer name	Sequence
hac1-1 F	CCGATTAGGCTGTTGCTG
hac1-1 R	TTCAGGAATGCAGGGTAAAG
hac1-3 F	TGAGGTGCAGCACAACTG
hac1-3 R	ATGTTGCACCCGCATAGT
hac1RNAm F	AGCATGGAGTTGCAGGAG
hac1RNAm R	AGGCAATTGCGAGTCTTG
hac2 F	TGGTCCAATAGGGCTGAG
hac2 R	TCCAGCTTTTCCATCAGG
hac4-1/4 F	GCATGTCAGGCCCAATAG
hac4-1/4 R	ACAGCAGCGTGTTGATGA
hac4RNAm F	CCCGGACAAACTTCACAG
hac4RNAm R	CGTTCCCACCAAGATTGA
hac5-7 F	TTTCTGATGCAACTGCTTGT
hac5-7 R	CGCGAACGTCAAAACTTC
hac5-8 F	GGAGTCACGGCATTATGTCT
hac5-8 R	CTAAGAGTACTGGCTGGCAA
hac5RNAm F	TTGCCATTGACAACATTCC
hac5RNAm R	GTTCTTGCTGGCAGATGG
hac12-2 F	CAAAATCCCCAAATCGAAG
hac12-2 R	CACCTAGCACCGTCAAGTG
hac12-3 F	CAATTGCGAGTCTTGCAGGT
hac12-3 R	AGAGATTACCCGCATCGAGG
hac12RNAm F	TGCCGTTCTCCAGTTTGT
hac12RNAm R	CACTCGGATTCCTTGCAG
haf1-3 F	GCTTCTGACGTTTCGACCTC
haf1-3 R	GGTTTTCAGAGGCAGCAGAG
haf1 RNAm F	GCGTTCCAAGATGTTTGC
haf1 RNAm R	AACTGCAACCCGAAATTG
haf2-2 F	GATACACATAGGCCATCATTCTGT
haf2-2 R	GTCAGTCATCTGTTGAAACAAACA
haf2-3 F	GAAAGGATCCGTCCTTGG
haf2-3 R	TGAGGGCTTCAAATCTGG
haf2 RNAm F	ATGGGAGCAATGATGAAGAG
haf2 RNAm R	AAAGGCTCGAGCATGTTGTT
CPK3 F	ATCTGGAGTGCTGGTGTGAT
CPK3 R	AATCCACGGATGATTTAGCA

E2Fb F	TGCCGATGAAAGAGGAAA
E2Fb R	GCTCGATGGAGTTTGTGG
E2Fc F	AGATGAGTTTTCCGCAACAG
E2Fc R	GCTCACCTCTGCATCTGACT
E2Fe F	TCACACTGAGCAGCGATTT
E2Fe R	TGGGTGACTTCGCTGAAC
LB SALK	GTCCGCAATGTGTTATTAAGTTGTC
SOC1 F	ATGAATTCGCCAGCTCCAAT
SOC1 R	GCTTCATATTTCAAATGCTGCA
FLC F	AGCCAAGAAGACCGAACTCA
FLC R	CATGATGATTATTCTCCATCTG
ChIP- E2Fb F	CCCTATGTTTCGAGGTTCCA
ChIP- E2Fb R	ACACGCGTTAAGTGTGAGCA
ChIP- E2Fc F	TCTAGCAATGCCGAGACTCC
ChIP- E2Fc R	ATTTGTTGGGCTTGGTCTTG
ChIP- E2Fe F	TCAAAGATAGACCCTGATTTTGC
ChIP- E2Fe R	GGGGATTTTGGTTTTGTAGGA
ChIP- FLC F	TCTCTGTGACGCATCCGTCG
ChIP- FLC R	CCCAGGTAAGGAAAAGGCG
