# Supplementary material for: HAC1 and HAF1 Histone Acetyltransferases Have Different Roles in UV-B Responses in Arabidopsis
Source: Front Plant Sci. 2017 Jul 10;8:1179. doi: 10.3389/fpls.2017.01179 (PMC5502275; doi:10.3389/fpls.2017.01179)
Supplement: Supplementary file 2 [file Image_1.PDF]

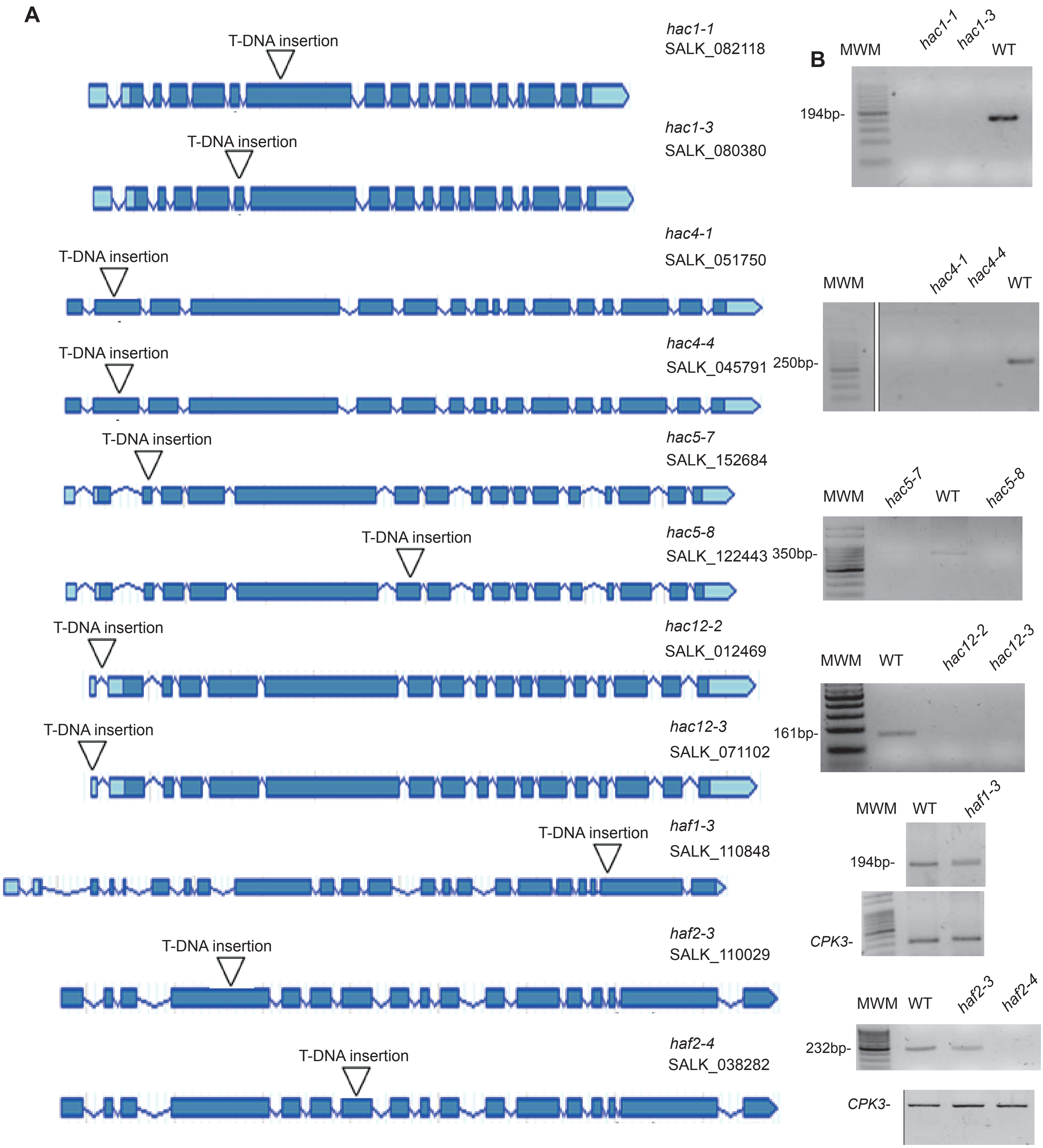

Figure S1. (A) Schematic view of *A. thaliana hac* and *haf* T-DNA insertional mutants. Blue boxes represent exons, thin blue lines represent introns, and light blue boxes the UTR regions. The location of the T-DNA insertions in the genes is shown and are represented as black triangles. **(B) RT-PCR analysis showing *HAC/HAF* transcript levels in WT and *hac/haf* mutants.** MWM: molecular weight markers. Molecular masses of the amplified PCR products are shown in the left.
