# Supplementary material for: HAC1 and HAF1 Histone Acetyltransferases Have Different Roles in UV-B Responses in Arabidopsis
Source: Front Plant Sci. 2017 Jul 10;8:1179. doi: 10.3389/fpls.2017.01179 (PMC5502275; doi:10.3389/fpls.2017.01179)
Supplement: Supplementary file 3 [file Image_2.PDF]

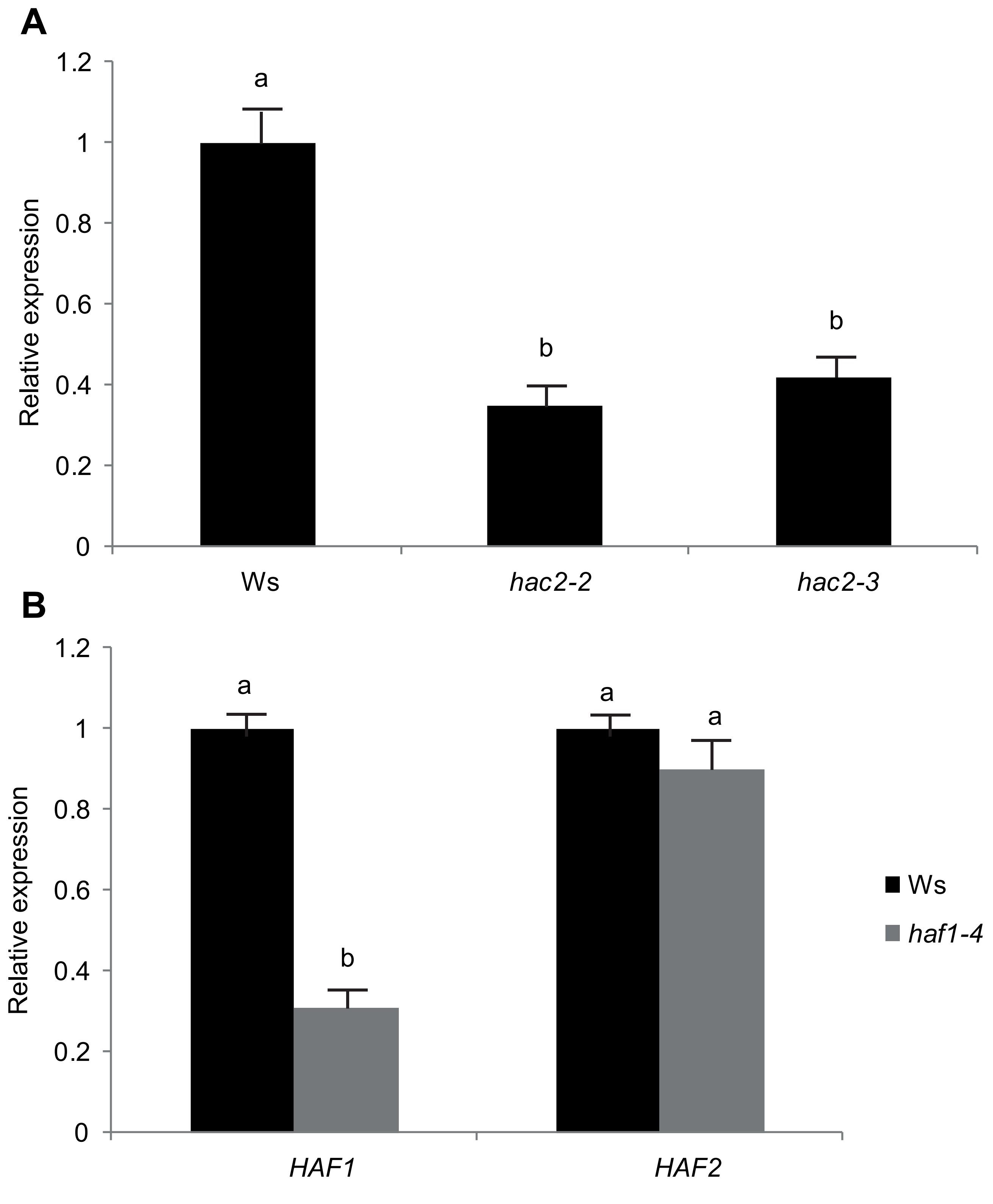

Figure S2. Relative expression of *HAC2* (A) and *HAF1* and *HAF2* (B) transcripts by RT-qPCR in WT and RNAi transgenic plants.

(A) *HAC2* transcript levels in Ws and *hac2-3* and *hac2-4* plants.

(B) *HAF1* and *HAF2* transcript levels in Ws and *haf1-4* plants. Expression values are relative to the *CPK3* control. Data show mean values ± S.E.M. of at least three independent experiments. Statistical significance was analyzed using ANOVA, Tukey test with $P < 0.05$; differences from the control are marked with different letters.
