# Supplementary material for: HAC1 and HAF1 Histone Acetyltransferases Have Different Roles in UV-B Responses in Arabidopsis
Source: Front Plant Sci. 2017 Jul 10;8:1179. doi: 10.3389/fpls.2017.01179 (PMC5502275; doi:10.3389/fpls.2017.01179)
Supplement: Supplementary file 4 [file Image_3.PDF]

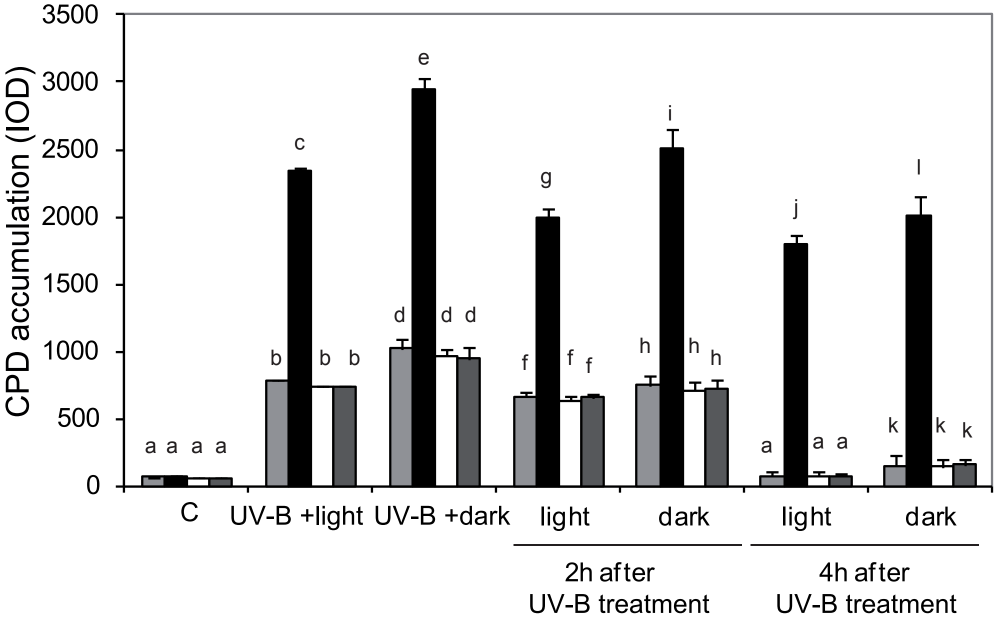

FIGURE S3. CPD levels in the DNA of WT Col-0 (light grey bars), *ham1-1* (black bars), *haf1-3* (white bars) and *hac1-3* (dark grey bars) plants under control conditions without UV-B, immediately after the UV-B treatment, or 2 h or 4h after a 4-h UV-B treatment. Experiments were done under conditions that allowed photorepair in the light or under dark conditions. CPD levels are indicated as integrated optical density (IOD) values. Results represent averages 6 SE of three independent biological replicates. Statistical significance was analyzed using ANOVA, Tukey test with $P < 0.05$; differences from the control are marked with different letters.
