# Supplementary material for: HAC1 and HAF1 Histone Acetyltransferases Have Different Roles in UV-B Responses in Arabidopsis
Source: Front Plant Sci. 2017 Jul 10;8:1179. doi: 10.3389/fpls.2017.01179 (PMC5502275; doi:10.3389/fpls.2017.01179)
Supplement: Supplementary file 5 [file Image_4.PDF]

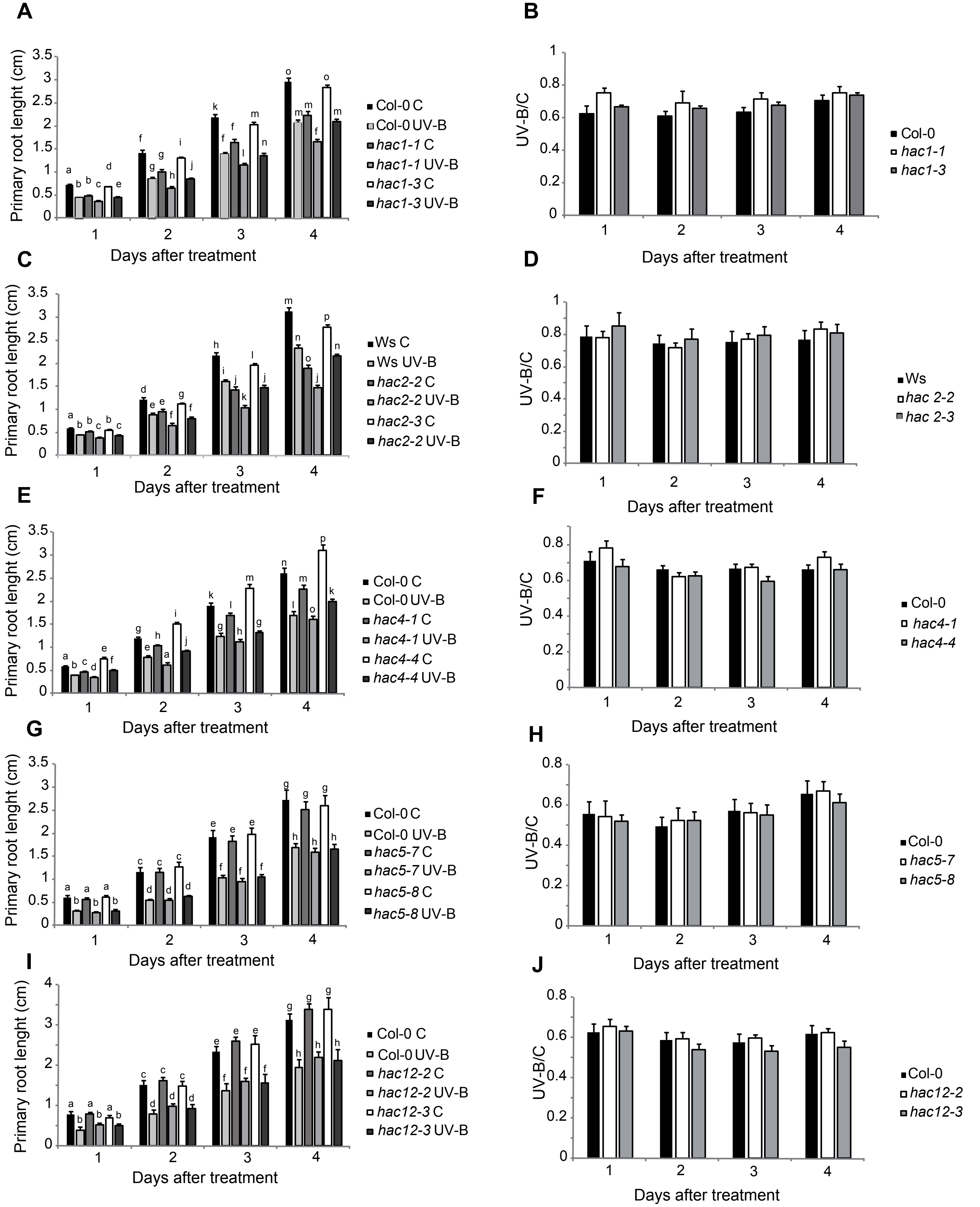

Figure S4. Primary root inhibition assays in WT and *hac* mutant plants after UV-B exposure.
Graph of average root lengths in Col-0 and *hac1* plants (A), Ws and *hac2* plants (C), Col-0 and *hac4* plants (E), Col-0 and *hac5* plants (G), and Col-0 and *hac12* plants (I) up to 4 days after a UV-B treatment or under control conditions in the absence of UV-B (C). Statistical significance was analyzed using ANOVA, Tukey test with $P < 0.05$; differences from the control are marked with different letters. The average root lengths after UV-B exposure relative to the length in control seedlings is shown in B, D, F, H and J; differences from the control are marked with different letters. Results represent the average of 20 biological replicates ± S.E.M.
