# Supplementary material for: HAC1 and HAF1 Histone Acetyltransferases Have Different Roles in UV-B Responses in Arabidopsis
Source: Front Plant Sci. 2017 Jul 10;8:1179. doi: 10.3389/fpls.2017.01179 (PMC5502275; doi:10.3389/fpls.2017.01179)
Supplement: Supplementary file 6 [file Image_5.PDF]

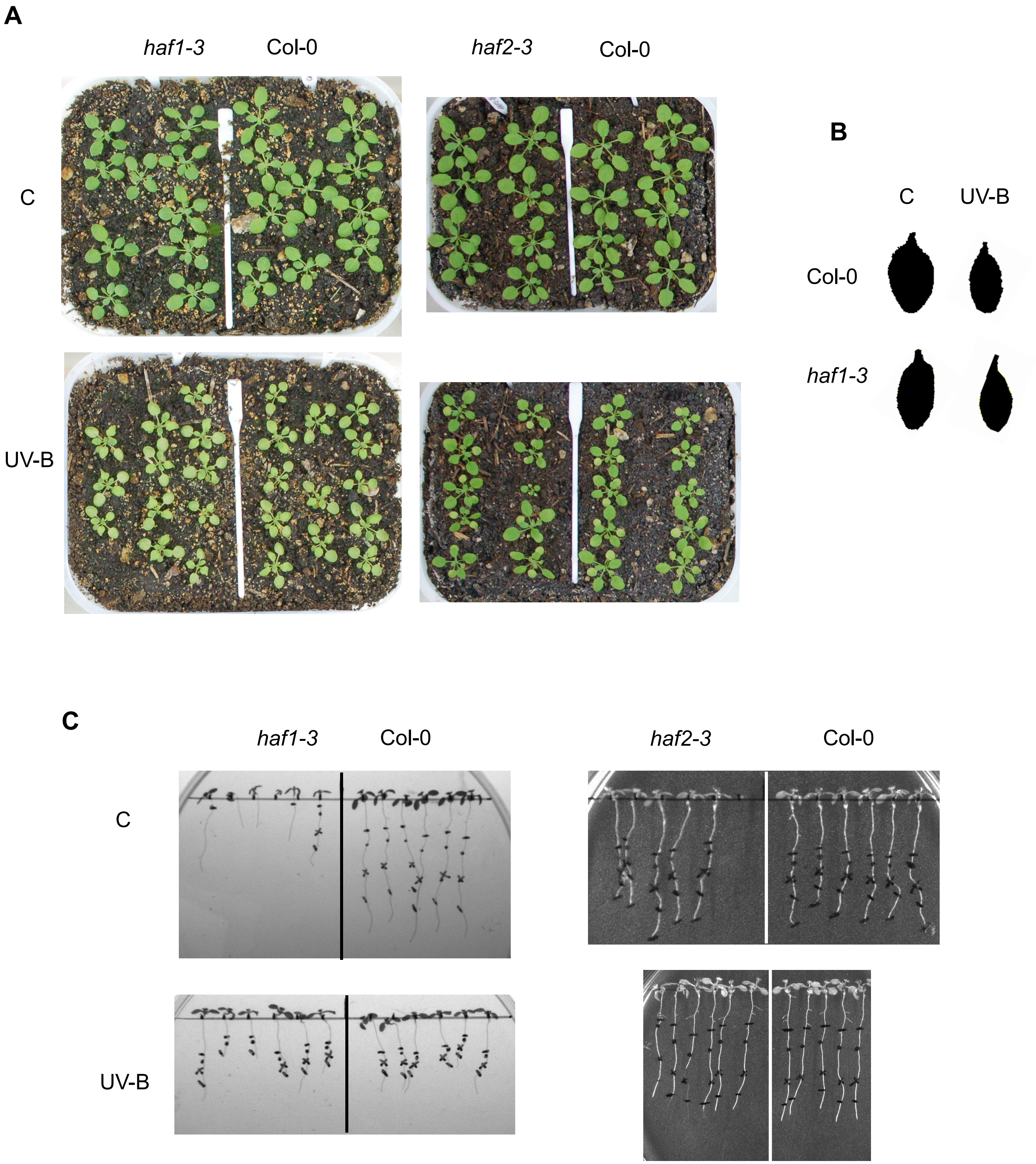

Figure S5. Representative pictures of *haf1* and *haf2* plants after UV-B exposure.
(A) Representative picture of Col-0 and *haf1-3* and *haf2-3* mutant plants that were treated with UV-B radiation for 4 h or were kept under conditions in the absence of UV-B, 15 d after the end of the treatment. C, control.
(B) Silhouettes of Col-0 and *haf1-3* leaf #5 detached from control and UV-B plants shown in (A).
(C) Representative picture of Col-0, *haf1-3* and *haf2-3* roots treated with UV-B radiation for 1 h or kept under conditions in the absence of UV-B 4 d after the end of the treatment.
