# Supplementary material for: HAC1 and HAF1 Histone Acetyltransferases Have Different Roles in UV-B Responses in Arabidopsis
Source: Front Plant Sci. 2017 Jul 10;8:1179. doi: 10.3389/fpls.2017.01179 (PMC5502275; doi:10.3389/fpls.2017.01179)
Supplement: Supplementary file 7 [file Image_6.PDF]

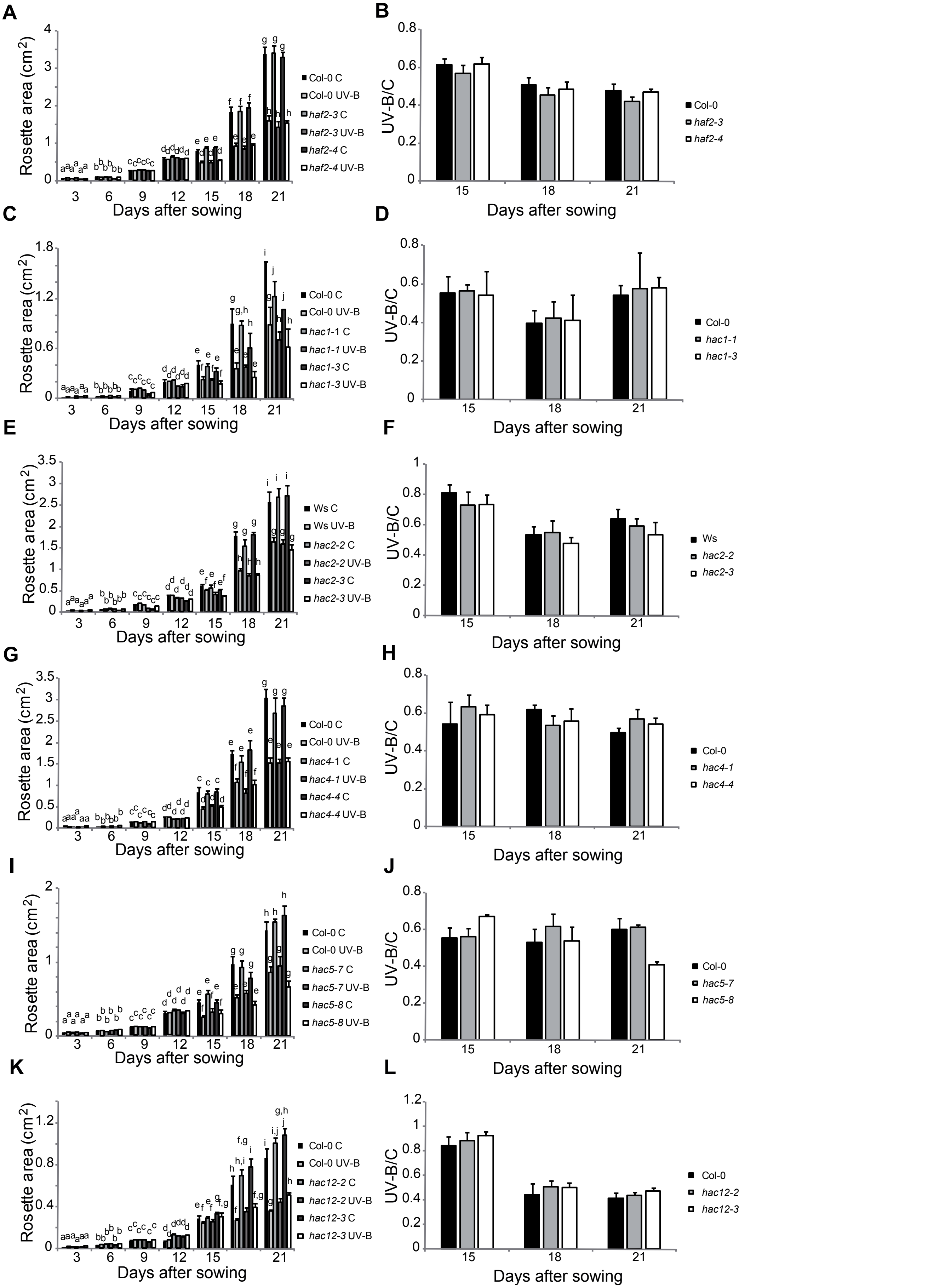

Figure S6. Plant growth inhibition by UV-B in *haf2* and *hac* mutants.

WT (Ws and Col-0) and *haf2* and hac plants were treated with UV-B radiation for 4 h (right) or were kept under conditions in the absence of UV-B. (A, C, E, G, I and K) Rosette area of control and UV-B treated Ws and *haf2* (A), Col-0 and *hac1* (C), Ws and *hac2* (E), Col-0 and *hac4* (G), Col-0 and *hac5* (I), and Col-0 and *hac12* (K) plants measured every 3 days from germination until 21 DAS. Plants were UV-B treated 12 DAS. (B, D, F, H, J and L) The ratio of rosette areas of UV-B treated vs control plants for each line is shown. Results represent the average of 10 biological replicates ± S.E.M. Different letters denote statistical differences using ANOVA, Tukey test with $P < 0.05$.
