# Supplementary material for: HAC1 and HAF1 Histone Acetyltransferases Have Different Roles in UV-B Responses in Arabidopsis
Source: Front Plant Sci. 2017 Jul 10;8:1179. doi: 10.3389/fpls.2017.01179 (PMC5502275; doi:10.3389/fpls.2017.01179)
Supplement: Supplementary file 8 [file Image_7.PDF]

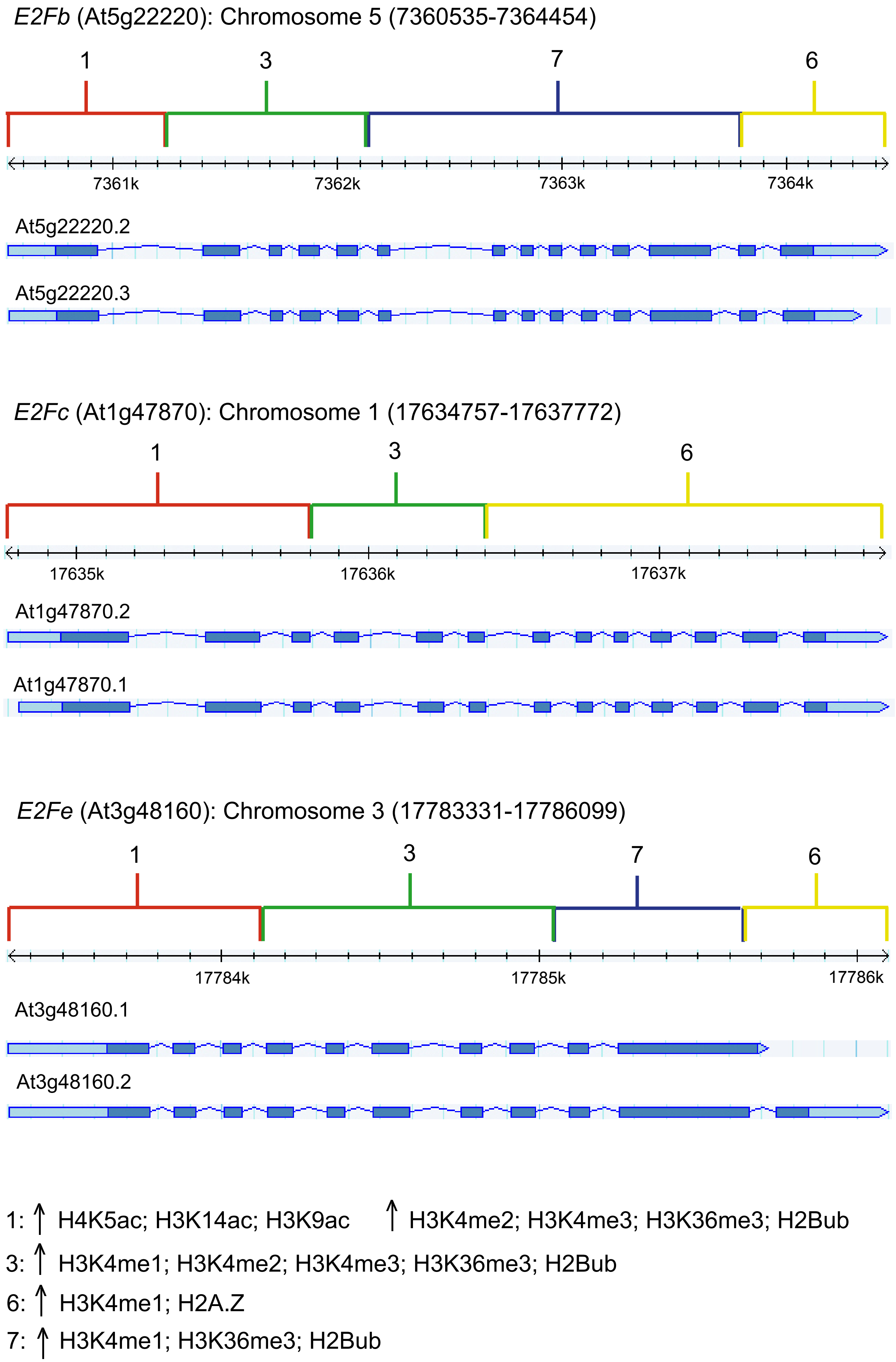

Figure S7. Prevalent chromatin states of *E2Fb*, *E2Fc* and *E2Fe* according to data published in Sequeira-Mendes et al. (2014).
